# Cross-model disagreement as a reference-free signal for prioritizing human review in medical speech transcription

**DOI:** 10.3389/frai.2026.1829902

**Published:** 2026-07-01

**Authors:** Abdolamir Karbalaie, Fernando Seoane, Farhad Abtahi

**Affiliations:** 1 Department of Clinical Science, Intervention and Technology, Karolinska Institutet, Stockholm, Sweden; 2 Department of Clinical Physiology, Karolinska University Hospital, Stockholm, Sweden; 3 Department of Textile Technology, Faculty of Textiles, Engineering and Business Swedish School of Textiles, University of Borås, Borås, Sweden; 4 Department of Medical Technologies, Karolinska University Hospital, Huddinge, Sweden; 5 Department of Biomedical Engineering and Health System, School of Engineering Sciences in Chemistry, Biotechnology and Health, KTH Royal Institute of Technology, Huddinge, Sweden

**Keywords:** ambient AI scribe, automatic speech recognition, clinical documentation, digital health, ensemble methods, health informatics, uncertainty quantification

## Abstract

**Introduction:**

Ambient AI scribes generate transcripts at scale, but routine quality assurance is constrained by the absence of human-verified reference transcripts in most deployment settings. We evaluated whether disagreement among heterogeneous automatic speech recognition (ASR) systems can serve as an informative signal for localizing transcription uncertainty, using a public English-language medical-speech corpus rather than clinical encounter recordings.

**Methods:**

Eight commercial and open-source ASR systems were applied to 50 medical-education audio clips (8 h 14 min). Multi-model outputs were aligned, and a leave-one-out consensus procedure was used to score per-model agreement while reducing circularity.

**Results:**

Disagreement across models was sparse and localized: 72.1% of positions showed strong agreement (7-8 systems concordant), whereas only 2.5% were high-risk positions with minimal agreement (0-3 systems). Low-agreement regions were systematically enriched for meaning-bearing lexical differences, defined as lexical mismatches after excluding punctuation, contraction, numeric, and filler variation. A single-annotator human-corrected (HC) validation layer showed that transcription errors increased monotonically with decreasing agreement. At an illustrative post hoc threshold, flagging positions where six or fewer systems agreed selected 28.6% of tokens while recovering 93.7% of single-annotator HC-verified errors on this proxy corpus.

**Discussion:**

These findings suggest that cross-model disagreement may help focus human review on a small number of likely error-prone transcript regions. However, agreement among all systems does not guarantee correctness, because shared errors may remain undetected by this approach. Validation on real clinical encounter data is required before operational deployment.

## 1 Introduction

Ambient artificial intelligence (AI) scribes that record patient encounters and generate visit notes are increasingly being deployed, motivated by expectations and early evidence that they may reduce documentation burden and improve clinician well-being (Lukac et al., 2025; Afshar et al., 2025; Tierney et al., 2024). A recent randomized trial of 238 outpatient physicians across 14 specialties and 72,369 patient encounters found that one ambient AI scribe application (Nabla) reduced documentation time by 9.5% versus usual care, while both evaluated systems showed potential improvements in burnout-related measures, though these secondary endpoints require confirmation in larger trials (Lukac et al., 2025). As healthcare systems adopt these tools at scale, the underlying automatic speech recognition (ASR) component becomes a key dependency for clinical documentation.

However, transcription reliability remains a persistent concern. Even as ambient AI scribes demonstrate efficiency gains, clinicians have reported that AI-generated notes occasionally contain clinically significant inaccuracies (Lukac et al., 2025). A recent policy analysis emphasized that such tools require ongoing vigilance and active physician review rather than passive acceptance (Dai et al., 2025). The operational challenge therefore extends beyond generating a transcript to prioritizing human verification, because clinically oriented ASR systems still exhibit non-trivial error rates in patient-clinician conversations (Tran et al., 2022). A reference-free triage signal—one that identifies which spans of a transcript warrant careful checking without requiring a human reference transcript—may help focus human review and reduce the burden of manual verification. In practice, such a signal would present a consensus transcript in which tokens are visually differentiated by agreement level, enabling the reviewing clinician to concentrate on a sparse subset of flagged spans rather than reading the full transcript. The intended workflow is therefore selective rather than exhaustive: review effort would be directed first to higher-uncertainty regions, with the specific trade-off between coverage and workload evaluated later in the study. More broadly, efficiency and safety concerns related to traditional speech-recognition-assisted documentation have also been reported in electronic health record workflows (Hodgson et al., 2017).

A major barrier to systematic quality assurance (QA) is that standard ASR evaluation assumes access to a human-verified reference transcript, typically to compute aggregate metrics such as word error rate (WER) (Tran et al., 2022). In many practical settings, especially at scale, human-verified reference transcripts are often unavailable, limiting routine auditing (Tran et al., 2022; Negri et al., 2014). This limitation is compounded by the fact that aggregate error metrics collapse heterogeneous phenomena, conflating meaning-bearing lexical substitutions with superficial differences such as punctuation or formatting, motivating error-type-specific and semantic evaluation in clinical ASR (Shor et al., 2023). Although some ASR systems expose confidence-like signals, commercial products differ in whether they provide token-level confidence or uncertainty primitives suitable for clinical review. Even where confidence measures are available, calibration can be imperfect particularly when models trained on one domain are applied to another and raw scores may not directly indicate where careful checking is warranted (Yu et al., 2011). Reference-free quality estimation methods have been proposed precisely to address scenarios in which neither human references nor reliable internal confidence are accessible (Negri et al., 2014).

Instead of comparing each transcript with an external gold standard, we treat multiple ASR systems as parallel readers of the same speech signal: agreement suggests transcript stability, whereas disagreement marks spans that may warrant review. However, agreement across systems may also reflect shared errors rather than correctness, imposing a fundamental ceiling on any disagreement-based approach. This concept aligns with classical ASR system-combination and lattice consensus frameworks, which operationalize agreement structure across competing hypotheses (Fiscus, 1997; Mangu et al., 2000). Here, rather than combining systems solely to minimize average error, we use *disagreement* as an explicit triage signal to localize where manual verification is likely to yield the highest safety value.

For this approach to be clinically useful, two conditions should be met. First, low-agreement regions should preferentially capture meaning-bearing disagreements rather than trivial surface-form variation; otherwise, such a tool could inflate review burden without commensurate value (Shor et al., 2023). Second, because ASR performance is known to vary with disfluency and demographic or linguistic variability, including accent-related effects and multilingual settings, any disagreement-based triage signal should be evaluated for robustness and equity across these sources of variability (Mujtaba et al., 2024; Zolnoori et al., 2024; Koenecke et al., 2020).

The objective of this study was to assess whether multi-ASR agreement can function as an informative, reference-free signal of transcription uncertainty for review of medical speech transcription. The central question is whether disagreement among independent ASR systems is (1) sparse, (2) localizable, and (3) enriched for meaning-bearing lexical divergence (lexical mismatches remaining after surface-form differences–punctuation, contraction, numeric format, and filler words–have been excluded), because these properties are required for a useful review triage signal. This is a methodological validation study using a reproducible public medical-speech dataset rather than a clinical deployment corpus. The primary analysis is reference-free, with human-corrected transcripts used only as a pragmatic external validation layer. We therefore treat the present dataset as a proxy domain for testing whether disagreement behaves like a useful triage signal, not as a substitute for patient–clinician encounter data. We addressed four research questions: (1) Are agreement-based uncertainty estimates stable under different reference-construction choices? (2) Are low-agreement regions systematically enriched for content-category disagreements? (3) How do agreement and risk profiles vary across accent groups (the primary demographic variable available in this corpus)? (4) To what extent are disagreement patterns associated with recording quality vs. accent-related variability (as an indirect proxy for linguistic variability)?

## 2 Materials and methods

### 2.1 Study design and data collection

We compiled an English-language medical speech corpus from publicly available YouTube videos selected to reflect medically dense monologic speech, including medical education narration and dictation-like speech, rather than conversational patient–clinician encounters. Each source video was segmented into approximately 10-min audio clips, yielding *N* = 50 clips as the primary unit of analysis. For each clip, we retained the audio segment and associated non-identifying metadata (i.e., accent label, speaking rate, and acoustic properties). This public corpus was chosen as a reproducible proxy domain for methodological validation, not as a deployment dataset for ambient documentation. Speaking rate (words/min) and word counts were derived from the Gemini Flash 2.5 transcript by dividing the transcript word count by clip duration (minutes). This measure is used as a descriptive proxy and is not a human reference transcript. The dataset follows prior practice of constructing publicly available, web-sourced speech corpora for ASR development and benchmarking (e.g., GigaSpeech and MediaSpeech) (Chen et al., 2021; Kolobov et al., 2021). Our corpus differs from these in domain content (medical education rather than general media or news) but shares the web-sourcing methodology. This public corpus was chosen as a reproducible proxy domain for methodological validation rather than as a deployment-like clinical dataset. Because it consists of prepared monologic speech rather than conversational patient-clinician encounters, findings from this corpus should be interpreted as proxy-domain estimates.

Ethical requirements were assessed in accordance with institutional policy for secondary analyses of publicly available materials. No participant recruitment, intervention, or direct interaction was performed. To minimize privacy risks, analyses were reported at the clip and subgroup levels, speaker names and identifying fields were excluded from analytical tables, and no protected health information or personally identifiable information was collected or analyzed.

### 2.2 Accent annotation and verification

Accent labels were obtained primarily from video metadata (e.g., speaker biography, affiliation, or stated region). To reduce labeling noise and harmonize categorization, we performed a secondary verification step using Google Gemini 2.5 Flash for metadata harmonization only, not for primary analysis. All Gemini outputs were manually adjudicated for discordant or low-confidence cases, and final accent annotations were mapped to a standardized taxonomy through deterministic rules. Additional details are provided in Supplementary method S1.

### 2.3 Audio extraction and standardization

All recordings were originally distributed as video files with heterogeneous audio encoding. For each video, the audio stream was demultiplexed and decoded, and a fixed-length analysis segment targeting 600 s was extracted (or the full duration if the source was shorter). All processed clips were stored as mono WAV at 16 kHz sampling rate and 256 kbps bitrate, ensuring that every ASR system received acoustically identical input. Detailed technical characteristics of the original source video audio and the standardized analysis segments are provided in Supplementary Method S2. All ASR systems were applied to the same pre-processed audio files, no audio preprocessing differences confound system comparisons.

### 2.4 ASR systems

Each clip was processed through eight distinct ASR systems to quantify transcription uncertainty via cross-model disagreement (Table 1). The ensemble spanned different architectural families and deployment contexts, chosen to increase hypothesis diversity across providers and model families. We intentionally included a legacy pair (Wav2Vec 2.0 and NeMo QuartzNet) to probe sensitivity to ensemble composition; however, we report reliability for a modern subset (Whisper Turbo v3, Whisper Large v3, Gemini Flash 2.5, Vox Mini, Speechmatics, MedASR) to reflect a more deployment-realistic ensemble. Exact model endpoints, version identifiers, and inference/processing settings are listed in Supplementary Tables S3, S4. All systems were run with default inference settings to reflect typical deployment without domain adaptation. Because disagreement is partly a property of ensemble composition, the resulting risk profiles should be interpreted for this specific heterogeneous panel rather than assumed to transfer unchanged to another set of models.

**Table 1:** Descriptive statistics of the evaluation corpus and ASR ensemble (*N* = 50 clips).

Category	Measure	Value
Audio corpus	Number of clips	50
	Total duration	29,635.10 s (≈ 8 h 14 min)
	Clip duration (mean ± SD)	592.5 ± 53.2 s
	Clip duration (range)	223.9–600.0 s
	Sample rate	16 kHz (all clips)
	Channels	Mono (1 channel; all clips)
	Bitrate	256 kbps (all clips)
	Loudness (mean ± SD)	−25.18 ± 5.43 dBFS (RMS proxy)
Speech content	Words per clip (mean ± SD)	1,560 ± 340
	Words per clip (range)	579–2,292
	Speaking rate (mean ± SD)	158.1 ± 30.9 words/min
	Speaking rate (range)	92–229 words/min
Speakers	Videos/speakers	50 (one speaker per clip)
	Gender	28 male (56%), 22 female (44%)
	Accent distribution	10 Singaporean, eight American, eight Australian, six British, three Indian, two Arabic, two New Zealand, plus one each of French, German, Japanese, Korean, Nigerian, Spanish, Norwegian, Swedish, South African, Canadian, Filipino
ASR ensemble	Systems (*n*)	8 (Gemini Flash 2.5, Vox Mini, Speechmatics, Whisper Turbo v3, Whisper Large v3, MedASR, Wav2Vec 2.0, NeMo QuartzNet)
	Total aligned tokens	696,204
	Evaluated aligned positions (Consensus mode)	76,398
	Evaluated token positions (HC alignment)	76,736

Word counts and WPM are derived from the Gemini Flash 2.5 transcript word count divided by the measured clip duration (not from a human reference transcript). Accent labels were derived from public metadata and standardized to a cleaned taxonomy after automated verification and manual adjudication. “Total aligned tokens” denotes the sum of normalized token counts across all eight ASR outputs and all clips. “Evaluated aligned positions (Consensus mode)” denotes the number of Consensus pseudo-reference token positions used as the denominator for agreement/risk-band analyses (excluding all-gap columns and positions where the reference token is a gap). “Evaluated token positions (HC alignment)” is the denominator used only for external validation against HC transcripts; it differs slightly from the Consensus-mode denominator (76,736 vs. 76,398; Δ = 338 positions, 0.44%) due to differences in reference definition and alignment scope between consensus-based evaluation and HC-referenced evaluation.

dBFS, decibels full scale; RMS, root mean square; SD, standard deviation.

### 2.5 Transcript normalization and tokenization

Before alignment, all transcripts underwent uniform preprocessing designed to reduce superficial orthographic variation while preserving lexical distinctions needed for downstream error typing. Transcripts were tokenized by splitting on whitespace. For alignment, the primary normalized representation consisted of lowercasing only; punctuation stripping and the contraction/numeric/filler resources were not applied before alignment. Instead, these additional representations and rule sets were used downstream to attribute punctuation-, contraction-, numeric-, and filler-related differences without collapsing them into the alignment input. The contraction and numeric mapping tables cover the most frequent forms observed in the corpus but are not exhaustive; tokens not matched by any mapping rule are passed through unchanged and classified as Content differences if they do not match the reference.

Each transcript was maintained in three parallel representations (raw, normalized, and stripped) so that superficial formatting variation could be separated from potentially meaning-bearing lexical divergence during error-type labeling. The complete normalization rules and mapping resources are provided in Supplementary Method S4, Supplementary Tables S5–S7.

### 2.6 Aligned transcript representation and pseudo-reference modes

We aligned the eight normalized model transcripts for each clip into a shared token coordinate system, yielding an aligned token matrix in which rows correspond to ASR systems and columns correspond to aligned positions. Unless otherwise stated, analyses are reported over evaluated aligned positions, excluding all-gap columns (i.e., positions where all systems contain a gap). We evaluated outputs under three deterministic reference-construction modes: (i) consensus (primary), which selects a consensus token at each aligned position by majority vote; (ii) centroid, which selects a single existing model transcript that is most central among hypotheses; and (iii) single-model reference, which treats a designated model output as the reference for sensitivity analysis.

Pairwise token-level alignments were computed using a global Needleman–Wunsch dynamic-programming algorithm with a scoring scheme designed to favor lexical matches while tolerating minor spelling variation: exact match = +10; fuzzy match = +5 (Levenshtein distance ≤ 2 for tokens longer than two characters); mismatch = −1; and constant gap penalty = −2. The fuzzy-match rule was used only as an alignment heuristic to stabilize local token placement and should not be interpreted as semantic equivalence, particularly for short edit-distance medical terms. Multi-sequence alignment was then constructed progressively: the first transcript initialized the alignment matrix, and each subsequent transcript was pairwise-aligned against a backbone sequence (the first non-null token per aligned position) extracted from the current matrix, expanding the matrix with null gaps as required. This procedure yields the aligned token matrix in which each column represents a shared position, enabling per-position voting, disagreement labeling, and majority-strength computation. Because the progressive build order and fixed tie-breaking rule are deterministic rather than order-invariant, the reported disagreement counts should be interpreted with respect to this specific alignment specification. Full alignment parameters, tie-handling rules, and mode-specific implementation details are provided in Supplementary method S5.

### 2.7 Jackknife (leave-one-model-out) consensus for per-model scoring

**Figure 1:**
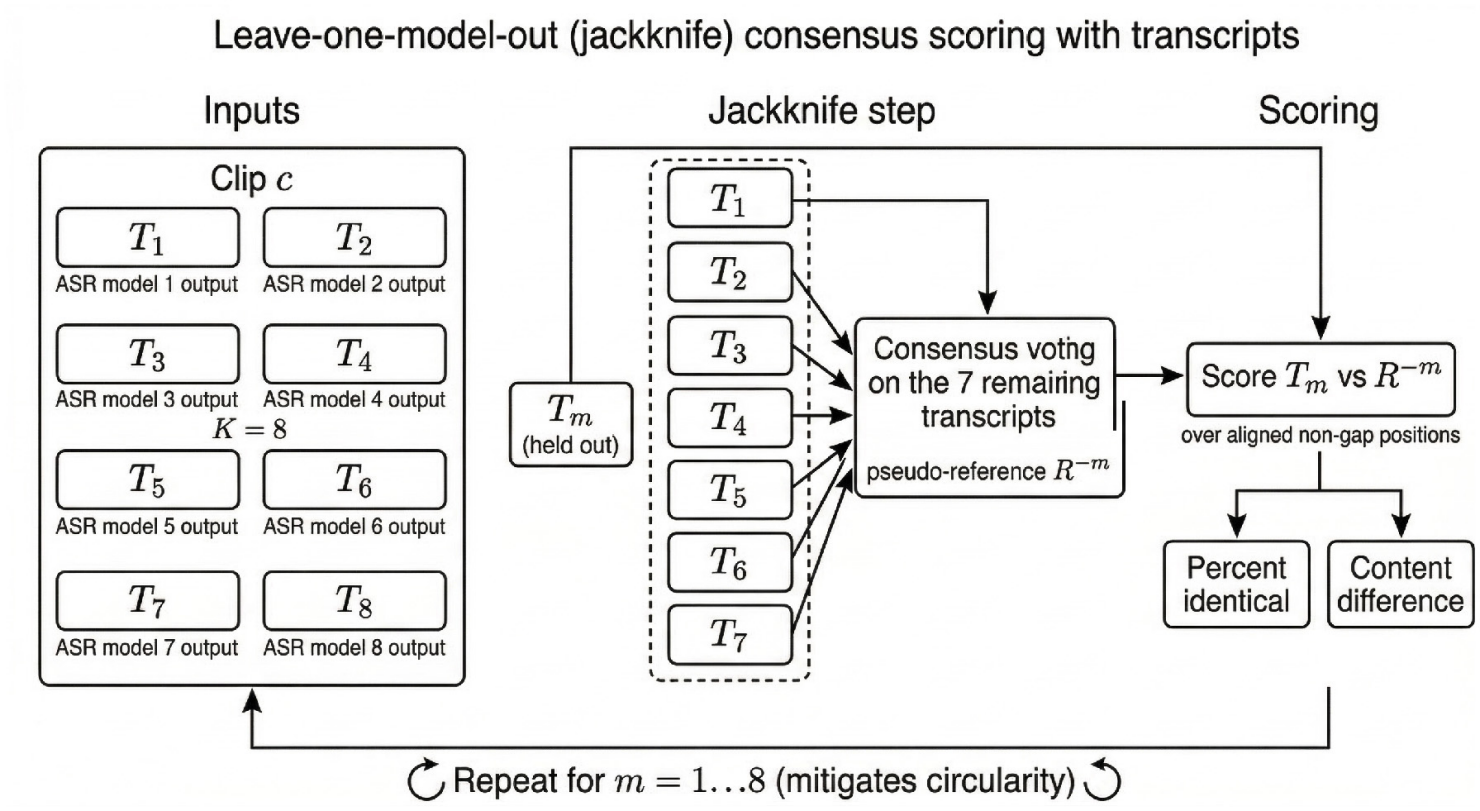
Leave-one-model-out (jackknife) consensus scoring for per-model evaluation (*K* = 8). For each clip *c*, eight ASR transcripts {*T*_1_, …, *T*_8_} are available. To score a given model *m*, its transcript *T*_*m*_ is held out and a pseudo-reference *R*^(−*m*)^ is constructed by applying the same consensus voting rule to the remaining *K*−1 = 7 transcripts {*T*_*j*_:*j*≠*m*}. Model *T*_*m*_ is then compared to *R*^(−*m*)^ over aligned non-gap positions to compute agreement metrics (percent identical and content difference). This procedure is repeated for *m* = 1, …, 8 to mitigate circularity when evaluating a model against an ensemble-derived pseudo-reference.

To mitigate circularity when scoring a model against an ensemble-derived pseudo-reference, we applied a jackknife (leave-one-model-out) procedure. For each clip and model *m*, we constructed a pseudo-reference *r*^(−*m*)^ by majority voting over the remaining *K*−1 = 7 hypotheses using the same Consensus voting rule (Supplementary Method S5). We then scored model *m* against its jackknife reference over non-gap reference positions using percent-identical and content-difference definitions (Figure 1).

### 2.8 Error taxonomy

For each clip and evaluation instance, we compared each model hypothesis to the selected reference token-by-token using the aligned transcript matrix. Each evaluated position was assigned exactly one label using a deterministic decision order, yielding a mutually exclusive taxonomy: (1) identical, (2) punctuation, (3) contraction, (4) numeric, (5) filler, and (6) content (all remaining lexical mismatches, including meaning-bearing differences). Full labeling rules and decision order are provided in Supplementary Method S6. Throughout, “content-category” disagreement denotes lexical category (not verified clinical correctness) and therefore supports enrichment analyses rather than accuracy claims. Finer clinical grading (e.g., distinguishing clinically equivalent paraphrases from genuine transcription errors) requires expert-adjudicated references and was therefore outside the scope of the present reference-free analysis.

### 2.9 Derived metrics and risk-band definitions

Using clip-level aligned comparisons to the selected reference (Consensus by default), we computed two primary metrics for each clip × model: percent-identical agreement (*p*_identical_) and content-difference rate (*r*_content_). Let *N*_total_ denote the number of evaluated aligned positions for a clip (reference token non-gap; excluding all-gap columns). We define these metrics in Equation 1 as:


$$\begin{array}{l} p_{\text{identical}}=\frac{N_{\text{ident}}}{N_{\text{total}}},\;r_{\text{content}}=\frac{N_{\text{content}}}{N_{\text{total}}}, \end{array}$$ (1)

where *N*_ident_ counts positions where the model's normalized token exactly matches the reference and *N*_content_ counts positions labeled as content differences under the disagreement taxonomy (excluding punctuation, contraction, numeric, and filler mismatches). Unless stated otherwise, corpus summaries are computed in two ways: (i) per-model, by averaging each model's clip-level metrics across clips; and (ii) as reference-mode “mean agreement,” by averaging percent-identical across models within each clip and then averaging across clips (reporting the standard deviation across clips).

To quantify ensemble support at each aligned position *i*, we computed majority strength *A*_*i*_ ∈ {0, …, 8}, defined as the number of ASR systems whose normalized token matches the reference token at *i* (gaps treated as non-matches). For 8 systems, we pre-specified three risk bands: low-risk *A*_*i*_ ∈ {7, 8}, medium-risk *A*_*i*_ ∈ {4, 5, 6}, and high-risk *A*_*i*_ ≤ 3. In the HC validation, threshold sweeps over *A*_*i*_ ≤ *t* were descriptive; the highlighted *A* ≤ 5 and *A* ≤ 6 operating points reported below are illustrative *post hoc* examples rather than pre-registered decision thresholds.

### 2.10 Human-corrected (HC) reference transcripts for external validation

To assess whether agreement-based risk bands correspond to actual transcription errors, we created a human-corrected (HC) reference transcript for each clip. A biomedical engineer with experience in medical terminology served as the sole annotator. This HC layer was intended as a pragmatic external validation set rather than a fully independent, adjudicated gold standard. For each clip, the annotator listened to the full audio recording and produced a transcript based on what was heard, consulting the consensus pseudo-reference only as a limited completeness check to reduce accidental omissions. Where audio-only disambiguation was insufficient, the annotator used non-identifying contextual cues from the source video (e.g., terms visible on slides such as institution names, medication names, or procedure labels) to resolve ambiguous tokens. The annotator was not informed of the risk-band assignments or the study hypotheses.

HC transcripts were used solely for evaluation of risk-band calibration and workload–recall trade-offs. They were not used to construct pseudo-references, define majority strength, or compute disagreement signals. All primary analyses remain reference-free in the sense that the proposed workflow and risk mapping can be computed using only cross-model outputs. The HC alignment denominator (76,736 evaluated token positions) differs slightly from the consensus-mode denominator (76,398) due to differences in reference definition and alignment scope (see Table 1 note). These design choices do not affect the reference-free risk map itself, but they reduce the independence of the HC evaluation and are considered in the interpretation.

### 2.11 Statistical analysis

Inter-model reliability was assessed by treating ASR systems as raters and clips as targets, computing single-measure intraclass correlation coefficients for absolute agreement (ICC[2,1]) separately for percent-identical and content-difference rate. In addition to the full ensemble, we report subgroup reliability for a modern subset (*k* = 6) and a legacy pair defined as Wav2Vec 2.0 and NeMo QuartzNet, to illustrate how agreement changes under different system compositions. Pairwise Spearman rank correlations were computed between per-clip score vectors for each model pair to identify systems with similar clip-wise behavior. We also computed mean paired differences in clip-level percent-identical between model pairs, defined as Δ*p*_identical_ = *p*_identical_(model(*m*))−*p*_identical_(model(*n*)) (percentage points), averaged over clips. Associations between clip-level audio covariates and disagreement signals were assessed using Spearman correlations, including correlations between clip duration, loudness, and estimated signal-to-noise ratio (SNR) proxies and the clip-level content-difference rate. Expanded reliability and correlation analysis details are provided in Supplementary method S7. Confidence intervals were computed for selected correlation analyses as described in Supplementary method S7.3. The HC workload-recall operating points are reported descriptively, clip-level bootstrap confidence intervals were not computed in the present analysis owing to the single-annotator design.

## 3 Results

### 3.1 Corpus characteristics

The evaluation corpus comprised 50 audio clips with a total duration of 29,635 seconds (approximately 8 h and 14 min). Mean clip duration was 592.5 s (SD = 53.2; range: 223.9–612.0). Mean speaking rate was 158.1 words per minute (SD = 30.9). The corpus included speakers from 18 accent categories, with Singaporean (*n* = 10), American (*n* = 8), Australian (*n* = 8), and British (*n* = 6) being most represented. Gender distribution was 56% male and 44% female. Descriptive statistics of the evaluation corpus and ASR ensemble are summarized in Table 1.

### 3.2 Per-model agreement with the Jackknife consensus pseudo-reference

**Figure 2:**
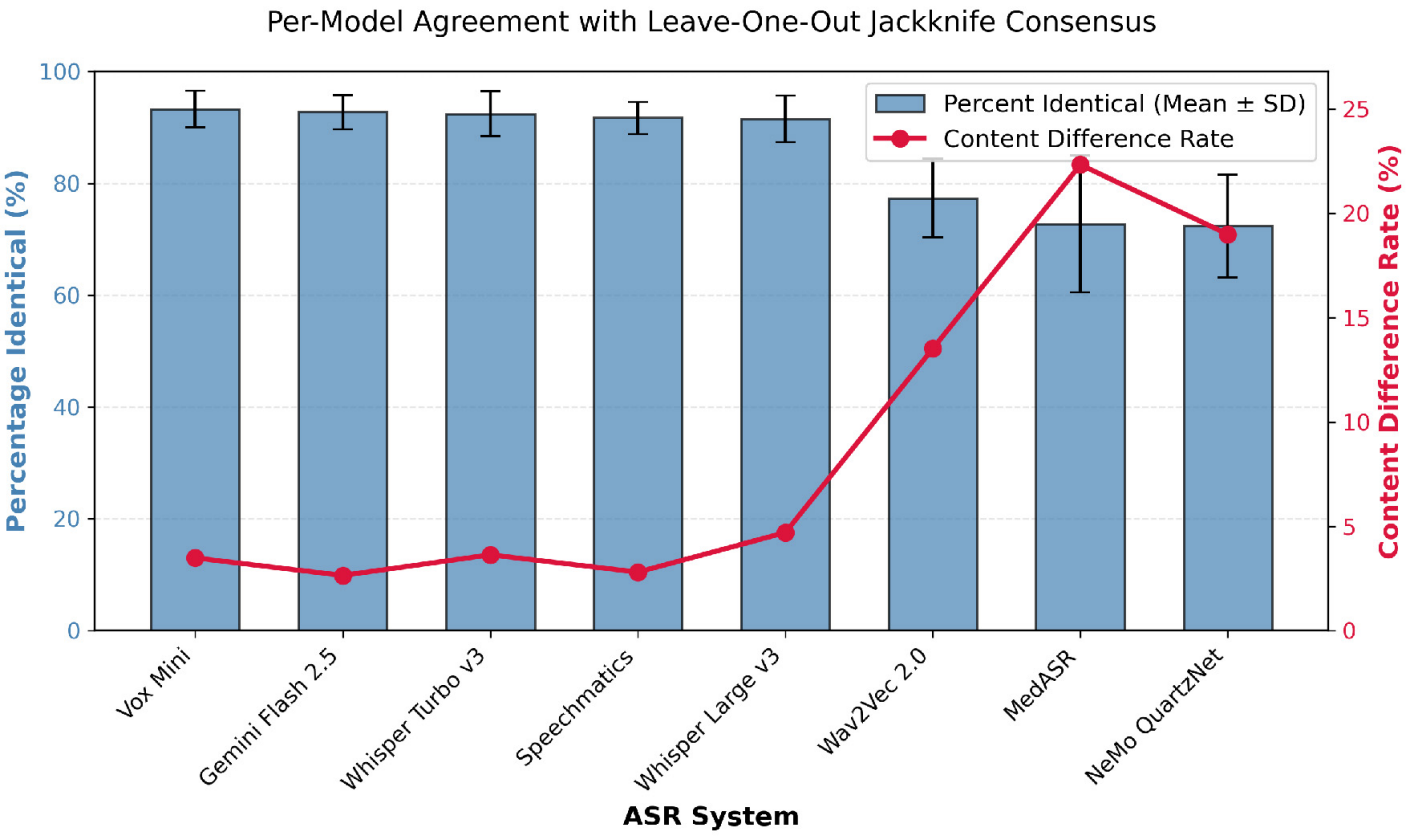
Per-model agreement under the jackknife consensus pseudo-reference. Mean percent-identical (±1 SD) across *N* = 50 clips, with an overlay showing mean content-difference rate per model. Higher bars indicate greater agreement with the ensemble-derived reference; lower content-difference rates indicate fewer meaning-bearing lexical divergences.

Mean percent-identical under jackknife (leave-one-model-out) consensus differed markedly across models (Table 2) and is visualized alongside content-difference in Figure 2. Vox Mini achieved the highest mean agreement (93.3%; SD = 3.2), whereas MedASR and NeMo QuartzNet showed substantially lower agreement (72.7%; SD = 12.2; and 72.4%; SD = 9.2, respectively). Notably, Gemini Flash 2.5 achieved the lowest content-difference rate (2.7%) despite ranking second in percent-identical, while Vox Mini led on percent-identical (93.3%) with a content-difference rate of 3.5%, indicating that the two metrics are not perfectly rank-correlated.

**Table 2:** Per-model agreement with jackknife (leave-one-model-out) consensus pseudo-reference.

ASR system	Percent-identical mean (SD)	Content-difference % mean (SD)
Vox Mini	93.3 (3.2)	3.5 (2.5)
Gemini Flash 2.5	92.7 (3.1)	2.7 (2.3)
Whisper Turbo v3	92.5 (4.0)	3.6 (3.1)
Speechmatics	91.7 (2.9)	2.8 (1.8)
Whisper Large v3	91.5 (4.2)	4.7 (3.7)
Wav2Vec 2.0	77.4 (7.0)	13.5 (7.6)
MedASR	72.7 (12.2)	22.3 (12.6)
NeMo QuartzNet	72.4 (9.2)	19.0 (10.2)

### 3.3 Winner analysis and inter-model reliability

Under jackknife evaluation, wins were distributed across four systems: Vox Mini (27/50; 54%), Gemini Flash 2.5 (13/50; 26%), Whisper Turbo v3 (7/50; 14%), and Whisper Large v3 (3/50; 6%). Winner counts stratified by accent group are shown in Supplementary Figure S1.

**Figure 3:**
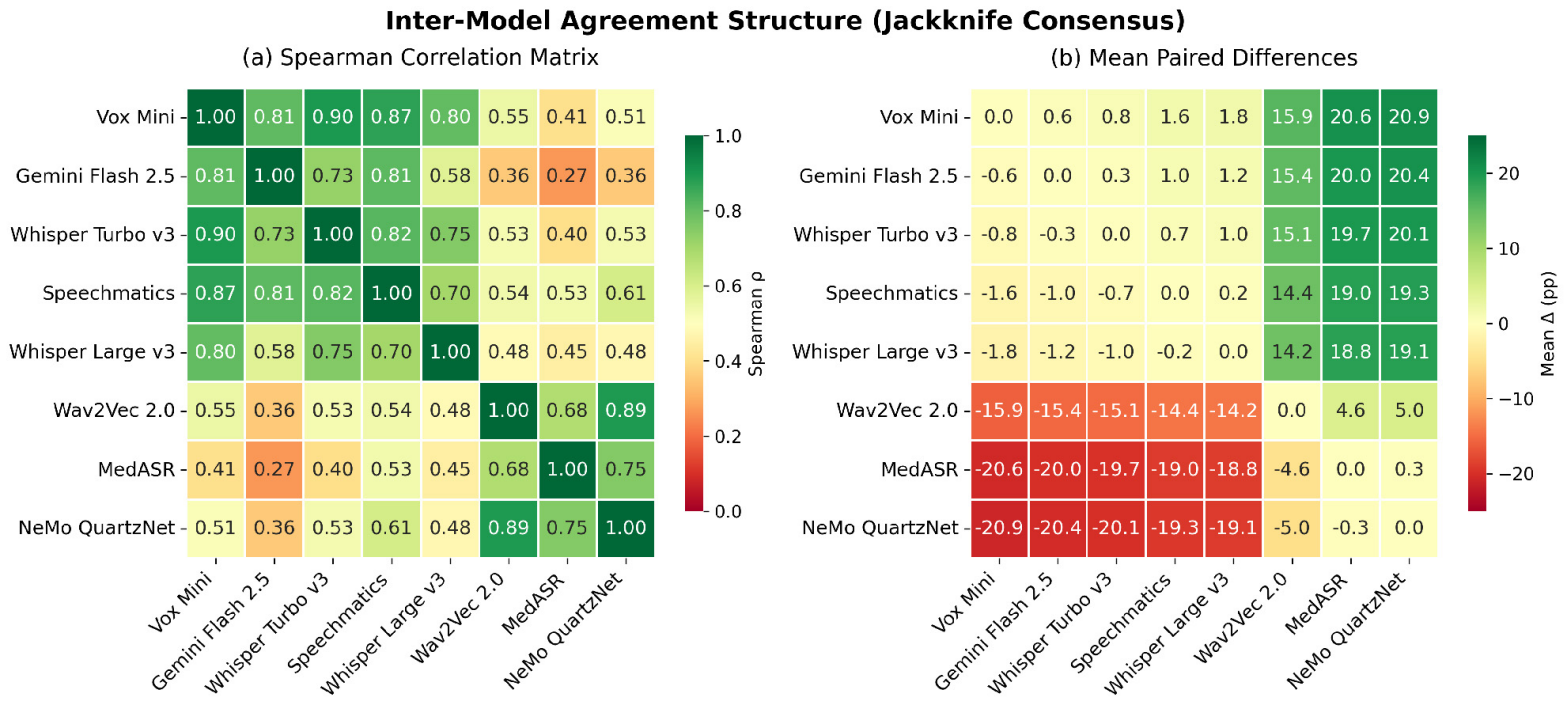
Inter-model agreement structure and systematic offsets in clip-level percent-identical performance (jackknife consensus scoring; leave-one-model-out). Panel **(a)** shows the pairwise Spearman rank correlation (ρ) matrix of per-clip percent-identical scores across the evaluated ASR models, computed over *N* = 50 clips under jackknife consensus scoring. Panel **(b)** shows the corresponding mean paired differences Δ in *p*_identical_ (percentage points), where each cell reports row − column averaged across the same clips; positive values indicate higher average *p*_identical_ for the row model. Diagonal entries are 1.00 in **(a)** and 0 in **(b)**.

Pairwise correlations in jackknife percent-identical varied substantially across model pairs (Spearman ρ range 0.27–0.90; median 0.56). Inter-model similarity structure and systematic percent-identical offsets are summarized in Figure 3. Absolute-agreement reliability remained low for the full ensemble (ICC[2,1] = 0.131 for percent-identical; ICC[2,1] = 0.155 for content-difference rate). Reliability was lower within the modern subset (*k* = 6; ICC[2,1] = 0.114 for percent-identical; 0.088 for content-difference rate) but high within the legacy pair (*k* = 2; ICC[2,1] = 0.715 and 0.703, respectively). The remaining four systems achieved zero wins each. Interpretation of this ICC pattern in the context of ensemble design is provided in Section 4.2

### 3.4 Majority-strength distribution

**Figure 4:**
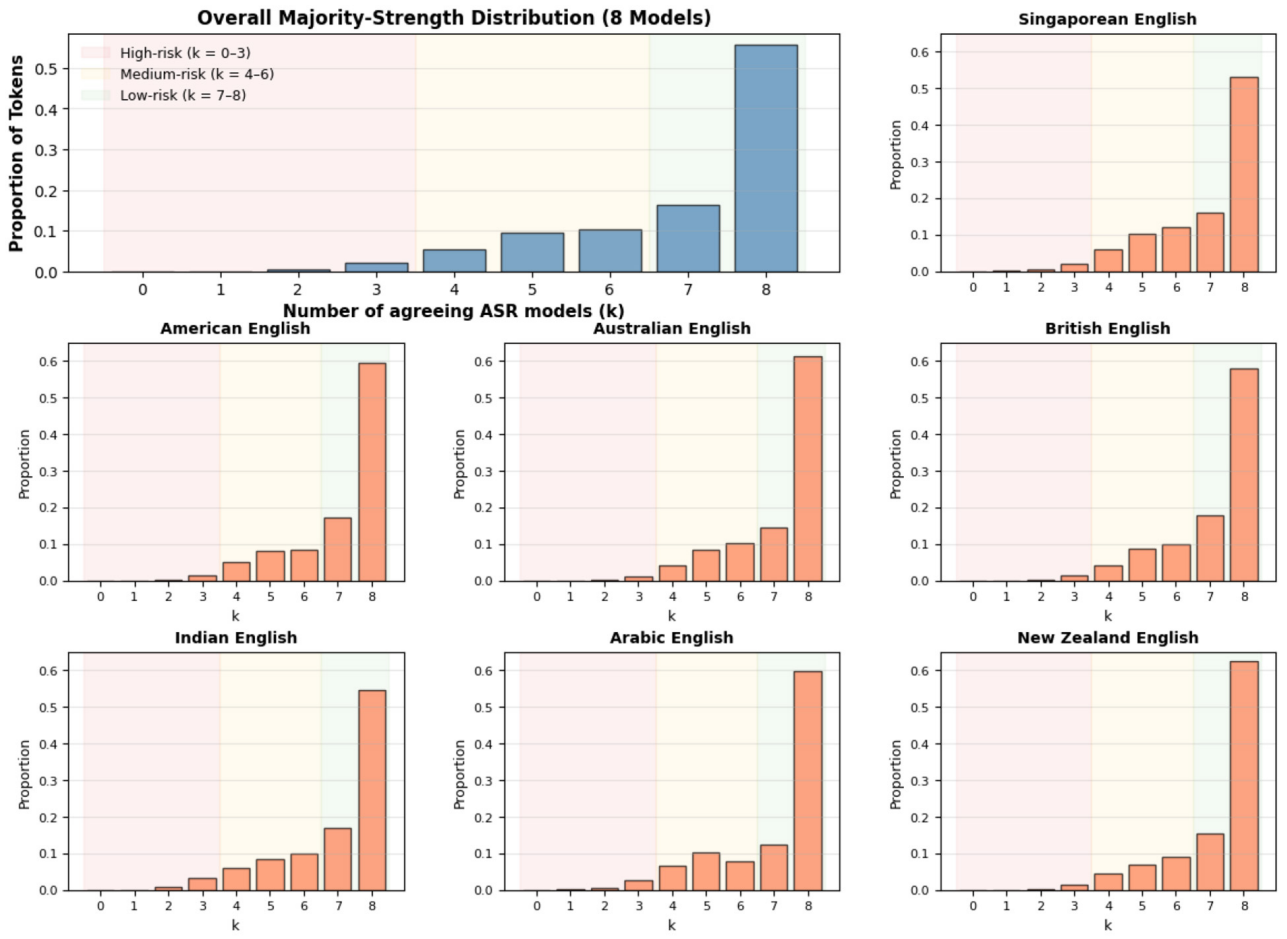
Majority-strength distributions overall and for selected higher-representation accent groups (*K* = 8; Consensus mode). Histograms show the distribution of agreement count *A*_*i*_ ∈ {0, …, 8}. Risk bands correspond to high (*A*_*i*_ ∈ {0, 1, 2, 3}), medium (*A*_*i*_ ∈ {4, 5, 6}), and low (*A*_*i*_ ∈ {7, 8}) uncertainty levels. Accent-stratified results are exploratory given small group sizes; panels for groups with a single clip (*n* = 1) represent individual recording characteristics and should not be interpreted as group-level estimates.

Given the observed variability in system behavior, we next examined whether disagreement is widespread or concentrated in a small subset of tokens. Among 76,398 evaluated aligned positions, the majority-strength distribution was concentrated at high agreement levels: 55.7% of positions achieved full agreement (*k* = 8), whereas positions with very low agreement were rare (*k* ≤ 2: 0.5%). Using the pre-defined risk-band mapping (high-risk: *A*_*i*_ ≤ 3), the corpus-level composition was 72.1% low-risk, 25.4% medium-risk, and 2.5% high-risk. The overall majority-strength histogram is shown in Figure 4.

Accent-stratified majority-strength distributions are illustrated in Figure 4, and accent-wise risk-band percentages with denominators (*n*_clips_, *n*_tokens_) are reported in Supplementary Table S8. Among higher-representation groups, low-risk proportions were 76.6% (American; *n* = 8), 75.9% (Australian; *n* = 8), and 69.2% (Singaporean; *n* = 10).

### 3.5 Content enrichment in low-agreement regions

**Figure 5:**
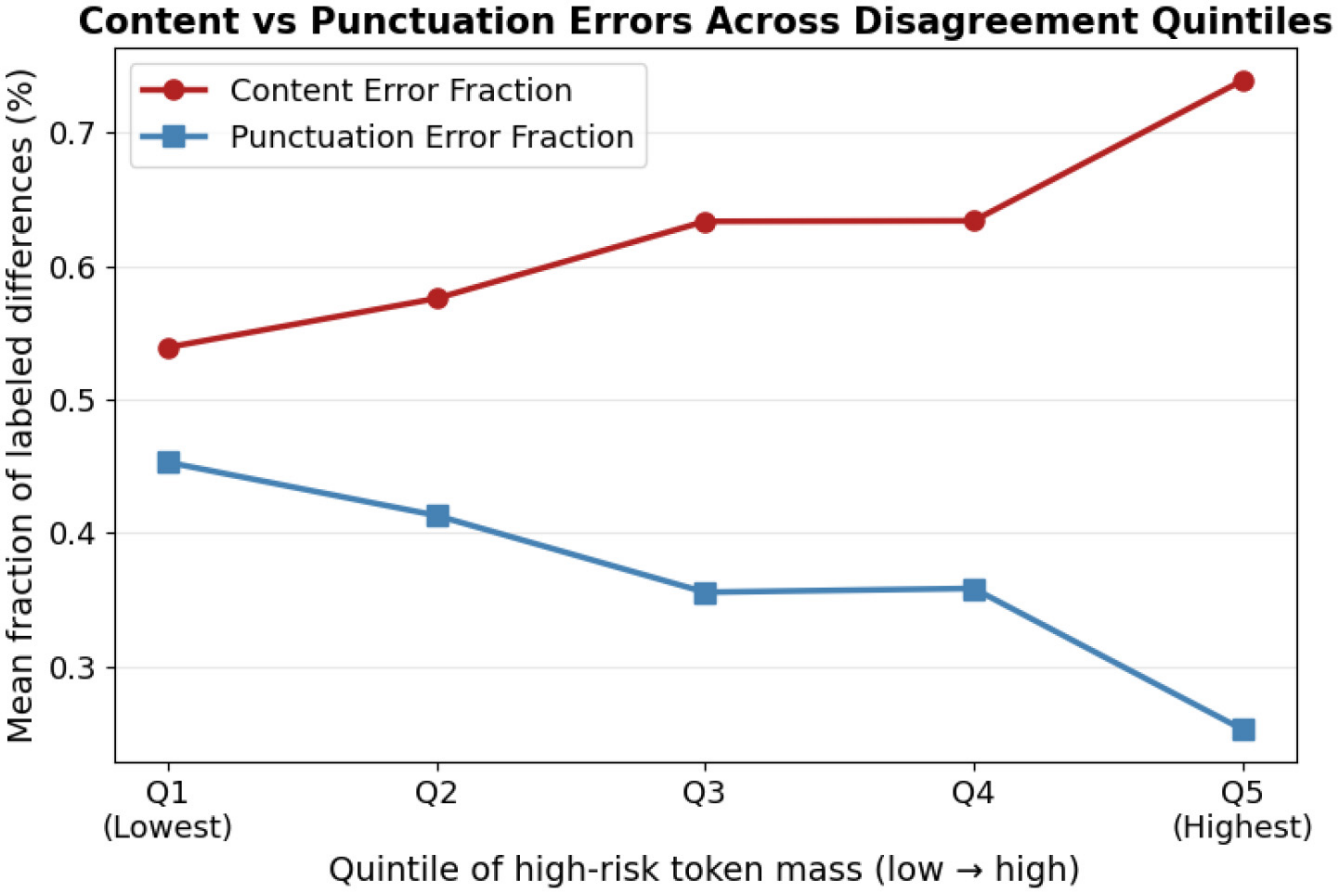
Disagreement composition across quintiles of high-risk token mass (Consensus mode; high-risk defined as *A*_*i*_ ≤ 3). Points show quintile means of the fraction of labeled differences attributed to Content and Punctuation categories. Quintiles are defined by high-risk token mass (percentage of evaluated aligned positions with *A*_*i*_ ≤ 3).

Disagreement composition differed across quintiles of high-risk mass (*p*_high_; Table 3). The mean content fraction increased from 53.9% in the lowest quintile to 73.9% in the highest quintile, whereas the mean punctuation fraction decreased from 45.3 to 25.3% (Figure 5). At the clip level (*N* = 50 clips), the positive association between *p*_high_ and content fraction [Spearman ρ = 0.559; 95% CI [0.333, 0.730]) spans weak to strong correlations, reflecting the limited sample size; the negative association with punctuation fraction was of similar magnitude (ρ = −0.547; 95% CI [−0.725, −0.315]). In absolute terms, *p*_high_ remained small even in the highest-risk quintile (mean 5.9%; maximum 11.4%).

**Table 3:** Disagreement composition by quintiles of high-risk mass.

Quintile	*N* clips	High-risk mass, % (range)	Content, %	Punctuation, %
1 (Lowest)	10	0.7 (0.3–1.1)	53.9	45.3
2	10	1.5 (1.3–1.6)	57.6	41.3
3	10	2.1 (1.8–2.4)	63.3	35.5
4	10	3.1 (2.5–3.5)	63.4	35.8
5 (Highest)	10	5.9 (3.6–11.4)	73.9	25.3

Quintiles were formed by ranking clips by high-risk mass *p*_high_ (Consensus mode; high-risk defined as majority strength *A*_*i*_ ≤ 3) and splitting into five equal-sized groups (*n* = 10 clips per quintile). “High-risk mass” is the percentage of evaluated aligned positions in the clip with *A*_*i*_ ≤ 3. “Content fraction” and “punctuation fraction” are computed within the set of labeled differences for each clip (fractions may sum to < 100% when other categories such as Contraction/Numeric/Filler are present) and are reported as quintile means.

### 3.6 Reference construction mode comparison

We compared the proposed leave-one-model-out (jackknife) consensus against three baseline reference methods: non-jackknife Consensus, Centroid, and Single-model references. Across clips, mean percent-identical agreement was 85.5% (SD 4.6%) under jackknife Consensus, 87.2% (SD 4.2%) under non-jackknife Consensus, 86.6% (SD 4.6%) under the Centroid reference, and 81.3% (SD 5.6%) under the Single-model reference. Against HC transcripts, mean agreement was 93.1% (SD 3.9%) for Consensus, 91.9% for Centroid, and 94.8% for a Single-model reference.

### 3.7 Clip-level covariate sensitivity

Finally, we examined whether disagreement patterns were associated with clip-level covariates. We computed a clip-level mean content-difference rate by averaging per-model content-difference rates across the ensemble and correlated this quantity with audio covariates. No covariate reached statistical significance. The largest-magnitude association involved speaking rate (ρ = −0.220; *P* = 0.125). Clip duration, loudness, and estimated signal-to-noise ratio showed near-zero associations (|ρ| < 0.07).

### 3.8 External validation against human-corrected (HC) transcripts

**Figure 6:**
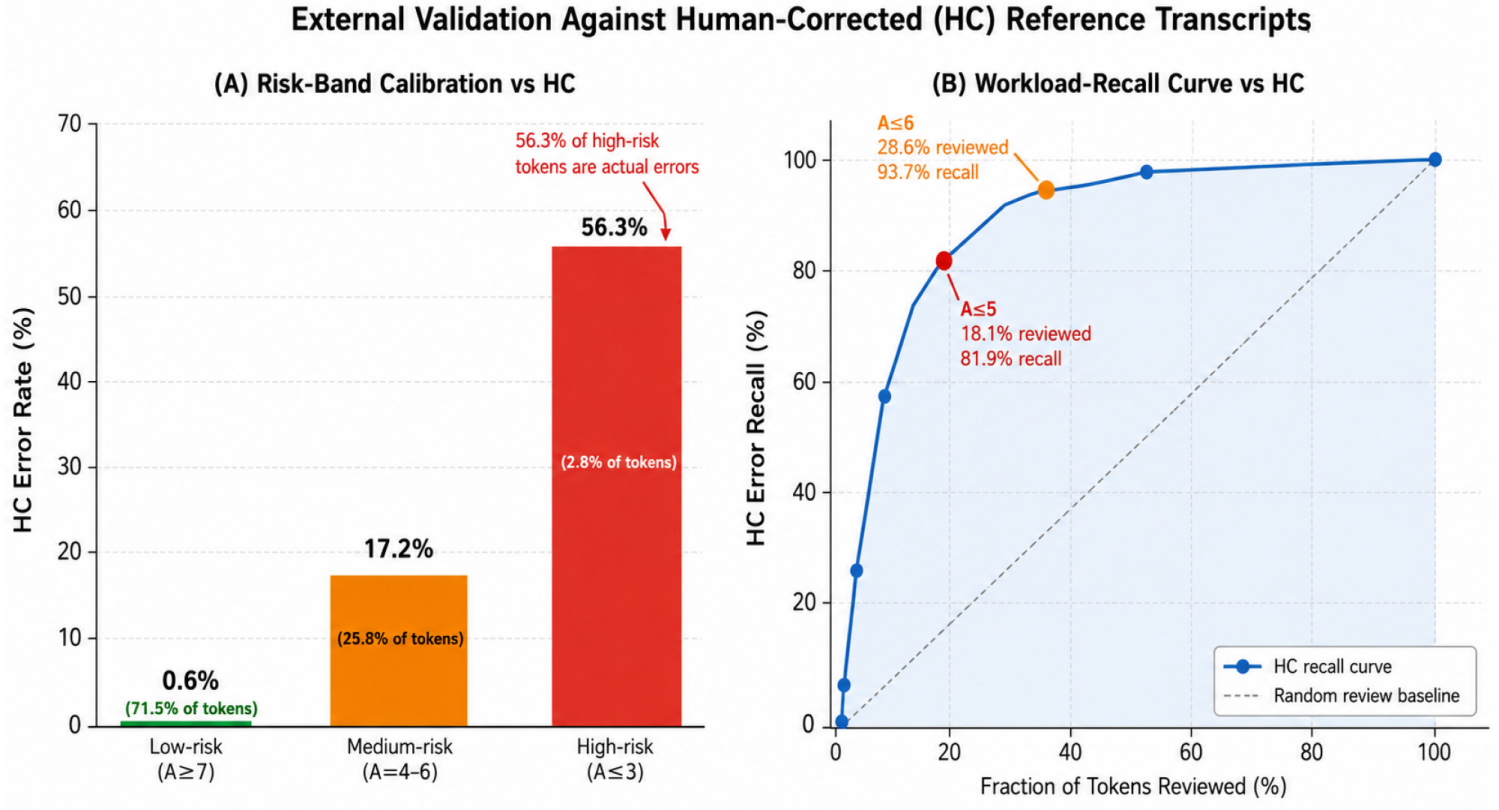
External validation of the disagreement-based risk map against single-annotator human-corrected (HC) reference transcripts (*N* = 50 clips; 76,736 HC token positions). **(A)** HC error rate by majority-strength risk band. Bars show the fraction of tokens in each band that are HC-verified transcription errors; token shares (% of all positions) are labeled inside bars. The monotone increase from low- to high-risk shows that HC-verified transcription errors were more common at lower majority strength on this dataset. **(B)** Workload–recall curve. The *x*-axis is the fraction of tokens sent for review at threshold *A* ≤ *t*; the *y*-axis is the fraction of all HC-verified errors recovered. Annotated operating points illustrate two post hoc thresholds (*A* ≤ 5 and *A* ≤ 6). The dashed diagonal represents random review. No confidence band is shown; clip-level bootstrap intervals are deferred to future work with larger corpora and multi-annotator references.

We evaluated the risk-band calibration against single-annotator HC transcripts across *N* = 50 clips and 76,736 aligned token positions (HC alignment denominator; see Table 1 note). Error rates increased monotonically across risk bands. Low-risk tokens (*A*≥7) had an HC error rate of 0.6% and comprised 71.5% of positions. Medium-risk tokens (*A* = 4–6) had an HC error rate of 17.2% and comprised 25.8% of positions. High-risk tokens (*A* ≤ 3) had an HC error rate of 56.3% and comprised 2.8% of positions (Figure 6A). The positive predictive value of the high-risk flag (*A* ≤ 3) was 56.3%, meaning approximately two in five flagged high-risk tokens were not HC-verified errors on this corpus. At the *A* ≤ 6 threshold, the estimated PPV was approximately 21%, reflecting the expected trade-off between broad coverage and review precision.

We next quantified review workload using descriptive threshold policies that flag tokens with agreement *A* ≤ *t*. The highlighted thresholds *A* ≤ 5 and *A* ≤ 6 are illustrative *post hoc* operating points, not pre-registered decision thresholds. Flagging tokens with *A* ≤ 5 selected 18.1% of positions and captured 81.9% of HC errors. Flagging tokens with *A* ≤ 6 selected 28.6% of positions and captured 93.7% of HC errors (Figure 6B). Finally, we quantified “shared-wrong” failures where all models agree with each other but disagree with HC. Among 4,914 HC errors, 78 occurred at *A* = 8, corresponding to a shared-wrong rate of 1.59%. Clip-level bootstrap confidence intervals were not computed for these operating points.

## 4 Discussion

### 4.1 Main findings

Taken together, the results support the central premise: cross-model disagreement is sparse enough to be useful as a triage signal and concentrated enough in meaning-bearing differences to substantially exceed random-flagging rates, though the modest positive predictive value (~56% at *A* ≤ 3) means that roughly two in five flagged high-risk tokens were not HC-verified errors on this corpus. These properties were demonstrated on comparatively clean audio single-speaker, prepared medical narration and the absolute prevalence of high-risk positions is expected to increase under real clinical encounter conditions, where turn-taking, ambient noise, and disfluency increase ASR difficulty. The practical implication is therefore a triage principle rather than a specific threshold: disagreement localizes uncertainty, and its composition shifts toward meaning-bearing lexical divergence as agreement decreases.

### 4.2 Clinical relevance and implications for ambient AI scribes

As ambient AI scribes are deployed at scale, the ASR component becomes an important dependency that can materially influence downstream note quality. A recent randomized trial demonstrated that such tools can reduce documentation time but also revealed that AI-generated notes occasionally contain clinically significant inaccuracies, underscoring the need for active physician oversight (Dai et al., 2025; Lukac et al., 2025). Similarly, a policy analysis warned of the risks of passive acceptance of AI-generated documentation and called for governance guardrails including disabling auto-accept features and requiring active review of diagnoses and billing elements (Dai et al., 2025). The present study does not evaluate full note-generation systems or patient–clinician encounters. Its contribution is narrower: it identifies a model-agnostic method for converting multiple transcript hypotheses into a risk map that localizes candidate spans for human verification.

In this context, low absolute-agreement ICC is consistent with the intended ensemble rationale, namely that systems fail on different clips rather than identically, although alternative explanations remain possible. High ICC within the legacy pair is consistent with architecturally similar systems sharing failure modes and contributing less independent signal to a disagreement-based ensemble. While including weaker or legacy systems can increase the quantity of disagreement, our modern-subset results still show low reliability, indicating that disagreement persists even among stronger systems and remains a viable triage signal. That said, risk-band prevalence and optimal thresholds are expected to change with ensemble composition and should therefore be recalibrated for the intended deployment configuration. As a sensitivity analysis, Consensus and Centroid reference construction produced similar clip-level summaries. The Single-model reference yielded the highest mean agreement to HC transcripts (94.8%), followed by Consensus (93.1%) and Centroid (91.9%). MedASR showed the lowest agreement and highest content-difference rate (Table 2) despite being domain-labeled, possibly reflecting domain/style mismatch with narrated medical education audio; disambiguating this requires expert-adjudicated references.

When clips were stratified by high-risk mass, the content fraction increased while punctuation decreased, indicating that lower-agreement regions were more often associated with meaning-bearing lexical differences than with superficial formatting variation. Even in the highest-risk quintile, high-risk tokens accounted for only a small share of positions (mean 5.9%), supporting the premise that a disagreement-based interface can direct attention to a limited subset of spans instead of requiring full-transcript review. In this workflow, the agreement threshold functions as a clinician-controlled review knob: inspecting only high-risk positions (*A* ≤ 3) restricts attention to a very small proportion of tokens, whereas expanding to high+medium positions (*A* ≤ 6) trades additional coverage for a still substantial reduction in review burden compared with full transcripts. This should be interpreted as a corpus-specific illustration of potential triage efficiency rather than as deployment-ready calibration. At the broader *A* ≤ 6 threshold, the estimated PPV was approximately 21%, meaning that the majority of reviewed tokens would not contain verified errors. Institutions considering deployment would need to weigh the high recall (93.7%) against this substantial false-positive burden, calibrating the threshold to the clinical context and the relative cost of missed versus unnecessary review.

### 4.3 Distinguishing lexical disagreement from HC-verified transcription error

A distinction must be drawn between content-category disagreement, defined here as lexical divergence in meaning-bearing word classes, and HC-verified transcription error. Our taxonomy classifies disagreements by lexical category rather than by clinical correctness. In the HC validation, 56.3% of high-risk tokens (*A* ≤ 3) were genuine transcription errors, compared with 0.6% of low-risk tokens (*A*≥7), indicating strong enrichment of verified errors in low-agreement regions. However, these estimates were obtained from a single annotator, without inter-rater reliability assessment, with limited use of the consensus transcript as a completeness check, on a public medical-education corpus and should not be treated as deployment-ready calibration. The pseudo-reference analysis supports enrichment of disagreement categories, whereas the HC layer provides limited corpus-specific error calibration. For example, disagreement between “hypertension” and “high blood pressure” would be classified as a content difference despite clinical equivalence, whereas “hypotension” in place of “hypertension” would represent a genuine transcription error. This explains why disagreement is informative but imperfect: flagged content differences may reflect true transcription errors, acceptable lexical variation, or alignment/normalization effects. Conversely, some errors will remain unflagged when all models converge on the same incorrect token. The 78 shared-wrong errors at *A* = 8 (1.59% of all verified errors) illustrate an important limit of the approach and motivate periodic random audit of high-confidence spans, a complementary safeguard that can be implemented without additional model infrastructure. These HC-based estimates were derived from the full 50-clip corpus, which included multiple accent groups, but the present data do not support accent-specific calibration because subgroup analyses were exploratory and unevenly sized.

### 4.4 Accent variability and robustness

Accent-stratified descriptive analyses indicated that disagreement burden may vary across accent groups, although these estimates should be interpreted with considerable caution: subgroup sizes were small, uneven, and insufficient to support firm conclusions. Prospective, adequately powered accent-stratified evaluation with expert-adjudicated references is essential before any equity-related claims can be made. A disagreement-based workflow could potentially make robustness gaps visible as differences in verification burden, offering a candidate approach for screening disparities in contexts where reference transcripts are unavailable, though validation against expert review remains necessary (Mujtaba et al., 2024; Zolnoori et al., 2024; Koenecke et al., 2020). The weak associations between audio quality covariates and disagreement metrics suggest that disagreement patterns reflect linguistic or phonetic difficulty more than gross signal degradation.

The present findings are consistent with the broader premise that model disagreement in medical AI can serve as a clinically informative signal rather than simply a design failure a principle appearing in recent ensemble approaches to medical AI (Abtahi et al., 2026; Fiscus, 1997; Mangu et al., 2000). The MEDLEY framework, as a recently published conceptual framework, proposes that medical AI systems should orchestrate multiple models while preserving their diverse outputs rather than collapsing them into a consensus (Abtahi et al., 2026). The central thesis that model imperfection can be reframed as a resource is consistent with our content-enrichment analysis: low-agreement regions preferentially capture lexical divergence in meaning-bearing categories. Our results provide an early empirical data point in support of this premise within the ASR domain, though on a proxy corpus rather than a clinical deployment setting.

### 4.5 Limitations

This study has several limitations. It is a methodological validation study rather than a clinical accuracy evaluation, and the primary analysis is reference-free, relying on pseudo-references and disagreement structure rather than human-verified gold-standard transcripts. The human-corrected layer, produced by a single annotator without inter-rater reliability assessment, therefore provides pragmatic external validation, and the main claim remains workflow-level rather than deployment-ready accuracy assessment. The dataset was also restricted to publicly available YouTube medical-education speech rather than ambient clinical encounters, which limits ecological validity and may affect disagreement prevalence and risk-band calibration in real-world use. In addition, the alignment and disagreement pipeline is partly design-dependent: the progressive alignment is deterministic but not strictly order-invariant, and the observed risk profile is influenced by ensemble composition. Accordingly, the reported findings should be interpreted for this proxy corpus and model panel, with recalibration required for other deployment settings. The comparatively clean audio conditions of prepared narration likely inflate agreement and compress risk-band prevalence relative to what would be observed in clinical encounters; accordingly, the reported workload–recall trade-offs represent optimistic estimates.

### 4.6 Future work

Future work should connect the risk map to external correctness through expert annotation of stratified subsets (e.g., a pre-registered sample of high-risk tokens and matched low-risk controls, ideally with two annotators and adjudication), enabling estimation of how strongly each risk band predicts verified errors and whether enrichment holds across speaker groups. Additional methodological work should test transcript-order permutations, exact-match-only alignment, and deployment-specific ensemble subsets to quantify how robust the risk map is to alignment and panel design. A clinician-centered usability study is also needed to test workflow claims directly (e.g., review time, correction yield, and perceived workload) when disagreement highlighting is integrated into routine documentation review. Scaling the corpus to include real clinical encounters with turn-taking and ambient noise would enable assessment of ecological validity and recalibration of risk-band prevalence under deployment-like conditions. Where access to real clinical encounter data is limited, complementary annotated or simulated dialogue corpora may support earlier-stage calibration and stress-testing under more realistic conversational conditions. In addition to real clinical encounter recordings, publicly available synthetic clinical dialogue datasets, such as the synthetic patient–physician conversation corpus of Fareez et al. (2022), offer a complementary resource for evaluating the disagreement-based workflow under conversational conditions with turn-taking and domain-specific terminology, without the access barriers associated with protected health data.

**Figure 7:**
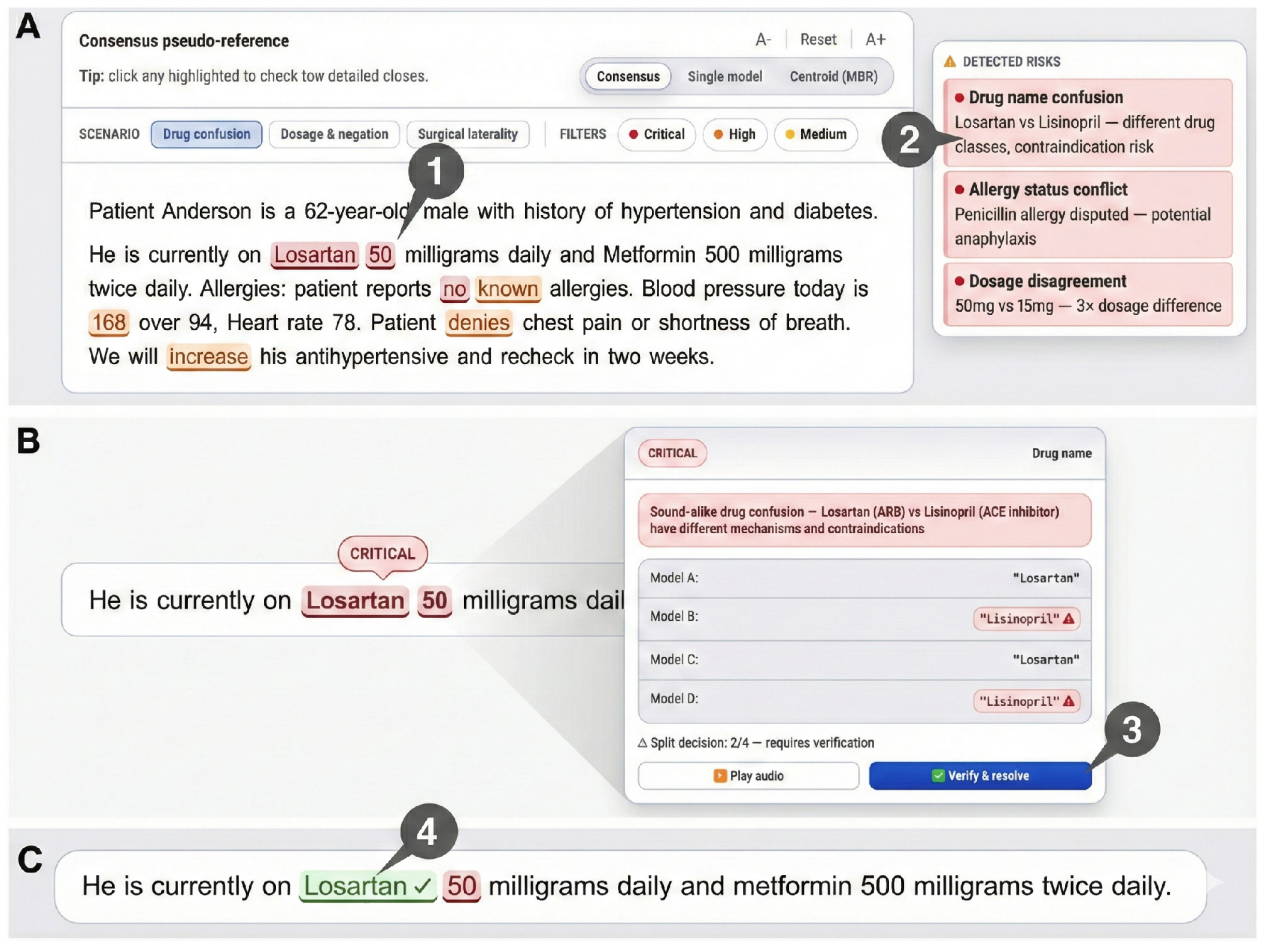
Translational design direction for a disagreement-based review workflow (ongoing follow-on work). The figure illustrates the intended interface and orchestration concept for applying the reference-free risk signal in practice. **(A)** Dashboard summarizing clip-level risk mass and a selectable review threshold alongside a risk-highlighted consensus transcript, allowing review effort to be concentrated on a sparse subset of spans. **(B)** Token inspection view exposing aligned alternatives from each ASR system with disagreement-category labels, enabling targeted adjudication where models diverge. **(C)** Review queue and audit panel supporting resolution, correction capture, and export for monitoring and threshold recalibration. Numbered callouts: (1) selected high-risk token, (2) linked risk-category item, (3) adjudication action with audit logging, (4) resolved indicator confirming closure.

To move from retrospective analysis toward deployable decision support, a follow-on implementation effort is currently underway to develop a risk-aware disagreement review interface and an “orchestrator” layer that treat multi-ASR outputs as complementary evidence rather than collapsing them into a single transcript (Figure 7). This ongoing work was not implemented or evaluated in the present study, Figure 7 is included to clarify the intended translational direction rather than to represent an evaluated system. The planned interface would visualize risk-banded consensus transcripts with per-model alternatives, while the orchestrator would route unresolved high-risk spans to additional checks and incorporate corrections to support threshold recalibration over time.

## 5 Conclusion

Cross-model disagreement may provide an informative, reference-free signal for localizing transcription uncertainty in medical speech. On this public medical-education corpus, disagreement was concentrated in a small minority of token positions and enriched for meaning-bearing lexical differences. A single-annotator HC validation layer indicated that disagreement-derived risk bands track transcription errors, with an illustrative threshold recovering 93.7% of HC-annotated errors while selecting 28.6% of tokens for review. The practical implication is not that disagreement replaces human oversight, but that it can make oversight more targeted and auditable by highlighting where review effort is most likely to matter. The primary unresolved validity question is whether risk-band calibration generalizes from narrated medical-education speech to real patient–clinician encounters with ambient noise, turn-taking, and domain-specific terminology.

## Data Availability

Analysis code and processed data files are available from the corresponding author upon reasonable request.
