# Supplementary material for: Cross-model disagreement as a reference-free signal for prioritizing human review in medical speech transcription
Source: Front Artif Intell. 2026 Jul 1;9:1829902. doi: 10.3389/frai.2026.1829902 (PMC13368871; doi:10.3389/frai.2026.1829902)
Supplement: Supplementary file 1 [file Data_Sheet_1.PDF]

Supplementary Material

1 SUPPLEMENTARY METHOD S1. ACCENT ANNOTATION WORKFLOW AND VERIFICATION

We obtained accent labels primarily from video metadata (speaker biography, affiliation, or stated region), which provided sociolinguistic context for each speaker. To reduce labeling noise and harmonize categorization across heterogeneous descriptions, we performed a secondary metadata-harmonization step using Google Gemini 2.5 Flash. Gemini was used only to map free-text metadata to a constrained list of accent categories and to provide a confidence estimate; it was not used for transcription, alignment, scoring, or any outcome analysis. Metadata labels were retained when concordant with a high-confidence Gemini suggestion; discordant or uncertain cases were resolved manually using the original metadata and contextual evidence. All final accent annotations were mapped to a standardized taxonomy through deterministic rules, converting granular descriptions into consistent categories to support cross-clip comparisons and exploratory subgroup summaries.

2 SUPPLEMENTARY METHOD S2. AUDIO EXTRACTION AND STANDARDIZATION

All recordings were provided as video files (e.g., MP4/WEBM) with non-uniform audio encoding parameters. For each source file (VIDxxx), the audio stream was demultiplexed and decoded using FFmpeg (v4.4.2), after which a fixed-length analysis segment targeting 600 s was extracted (or the full duration for sources shorter than 600 s). When required, the segment window was offset within the recording to avoid introductory material while retaining an approximately 10-minute analysis chunk; realized start offsets for the analyzed clips are summarized in Table S1.

To ensure identical ASR inputs across systems, all extracted segments were downmixed to mono (left/right averaging), resampled to 16 kHz, and exported as uncompressed WAV (16-bit signed PCM), yielding a final bitrate of 256 kbps (16 bits/sample $\times$ 16,000 Hz $\times$ 1 channel). No clipping mitigation, amplitude normalization, or silence-based trimming was applied, to preserve relative loudness differences and avoid preprocessing-induced segment alterations. Prior to transcription, each output file was verified for duration, sampling rate, and channel count.

Clip-level technical properties (duration, sample rate, bitrate, channels) and acoustic quality proxies (e.g., RMS level, peak level, crest factor, silence ratio; loudness and estimated SNR where applicable) are reported in Table S1 based on the standardized ASR input segments. The corresponding characteristics of the original audio streams extracted from the source containers are reported in Table S2.

3 SUPPLEMENTARY METHOD S3. ASR SYSTEMS – ARCHITECTURAL DIVERSITY AND PROVENANCE

Eight ASR systems were selected to span different architectural families and deployment contexts (commercial APIs, open-source models, and medical-domain engines). This diversity was essential to ensure heterogeneous failure modes and thereby render disagreement signals informative rather than redundant.

Table S1. ASR input segment audio characteristics (summary; mean ± SD [min, max]).

Variable	Summary (mean ± SD [min, max])
Number of clips (N)	50
Segment start (s)	4.96 ± 12.98 [0.00, 72.00]
Segment end (s)	597.42 ± 55.56 [223.00, 672.00]
Target duration (s)	592.46 ± 53.32 [223.00, 600.00]
Actual duration (s)	592.46 ± 53.32 [223.00, 600.00]
Sample rate (Hz)	16,000
Channels	1
Bitrate (bps)	256,000
RMS level (dBFS)	−25.18 ± 5.43 [−35.67, −13.56]
Peak level (dBFS)	−3.50 ± 3.73 [−14.12, 0.00]
Crest factor (dB)	21.68 ± 4.44 [13.56, 32.96]
Silence ratio	0.310 ± 0.174 [0.003, 0.924]

Note. Values describe the standardized 10-minute analysis segments fed to the ASR systems. dBFS, Decibels full scale; SD, Standard deviation.

Table S2. Original video audio stream characteristics (summary; mean ± SD [min, max]).

Variable	Summary (mean ± SD [min, max])
Duration (s)	922.08 ± 319.41 [223.91, 1661.71]
Sample rate (Hz)	44,100 ± 0
Channels	2 ± 0
Bitrate (bps)	320,466 ± 118,853 [103,339, 591,412]
Codec (mode)	AAC

Note. Values describe the original audio streams as obtained from the video containers before standardization.

Table S3. ASR systems: identifier mapping, exact model/endpoint strings, and provenance.

Internal ID	Display name	Exact model identifier	Provider	Type	Domain
GeminiFlash25	Gemini Flash 2.5	`gemini-2.5-flash`	Google	API	General
VoxMiniAPI	Vox Mini	`voxtral-mini-latest`	Mistral	API	General
Speechmatics	Speechmatics	`ENHANCED` (English)	Speechmatics	API	General
WhisperLargeV3	Whisper Large v3	`large-v3`	Open-source	Local	General
WhisperTurboV3	Whisper Turbo v3	`faster-whisper-large-v3-turbo-ct2`	Open-source	Local	General
MedASR	MedASR	`google/medasr`	Google (HF)	Local	Medical
NeMo_QuartzNet	NeMo QuartzNet	`QuartzNet15x5Base-En`	NVIDIA	Local	General
meta_wav2vec2	Wav2Vec 2.0	`wav2vec2-large-960h-lv60-self`	Meta (HF)	Local	General

Table S4. ASR inference and processing parameters.

ASR system	Chunk (s)	Overlap	Decoding mode	Beam	Diarization
Gemini Flash 2.5	∼600	None	Not configurable	N/A	Off
Vox Mini	∼600	None	Not configurable	N/A	Not configurable
Speechmatics	∼600	None	Not configurable	N/A	Off
Whisper Large v3	∼600	None	Beam search	5	Off
Whisper Turbo v3	∼600	None	Beam search	5	Off
MedASR	∼600	None	Greedy	N/A	Off
NeMo QuartzNet	∼600	None	Greedy	N/A	Off
Wav2Vec 2.0	∼600	None	Greedy	N/A	Off

Note: All ASR systems were run on the same pre-segmented ∼10-minute clips (≈600 s) with no overlap between segments. Model-specific decoding controls vary by provider: some local models expose decoding (e.g., beam search), whereas several APIs do not expose beam/temperature/top-k/top-p controls to the user. “Punctuation handling” reflects each system’s native output.

4 SUPPLEMENTARY METHOD S4. TRANSCRIPT NORMALIZATION AND TOKENIZATION

Before alignment, all transcripts underwent uniform preprocessing to reduce superficial orthographic variation that could otherwise inflate apparent disagreement. Each transcript was tokenized using whitespace boundaries (`str.split()`), producing a sequence of tokens. Hyphenated compounds (e.g., "twenty-one"), unit strings (e.g., "ng/mL"), and other multi-character sequences without internal whitespace were preserved as single tokens.

Three parallel token representations were maintained for each token to support different analytical purposes:

1. **Raw text (text):** the original token output by the ASR system, preserving all orthographic features (e.g., "Don't").
2. **Normalized (normalized):** the token after lowercasing only (e.g., "Don't" → "don't"). Internal punctuation (apostrophes), hyphens, and symbols are preserved.
3. **Stripped (stripped):** the normalized token with punctuation removed using the pattern `[.,!?;:'"()]` (e.g., "don't" → "dont").

Alignment and pseudo-reference construction (Methods S5–S6) operate on the normalized representation. Two tokens are treated as identical under exact-match alignment if their normalized forms match; near-matches may also align under the fuzzy-match criterion defined in Method S5. Error-type labeling (Method S6) leverages all three representations: punctuation-only differences are identified when normalized forms differ but stripped forms match; contraction differences are detected via deterministic contraction mappings applied after lowercasing (Table S6); numeric equivalence is detected using the raw token form together with the number-word mapping resources (Table S7); and filler words are identified using a curated lexicon matched on the normalized form (Table S5). All remaining mismatches not attributable to these categories are labeled Content.

Of note, the contraction/numeric/filler resources define equivalence classes for error attribution, not preprocessing steps applied prior to alignment. This multi-representation architecture ensures that superficial formatting variation (case and punctuation) can be separated from potentially meaning-bearing lexical divergences when summarizing disagreement.

Table S5. Filler expressions treated as non-lexical discourse markers during preprocessing and error typing.

**Filler lexicon (collapsed)**
um; uh; er; ah; erm; mm-hmm; hmm; eh; well; so; like; you know; i mean; sort of; kind of

Notes: Fixed *a priori*; matched case-insensitively after normalization.

5 SUPPLEMENTARY METHOD S5. MULTI-MODEL ALIGNMENT AND PSEUDO-REFERENCE CONSTRUCTION

5.1 S5.1 Multi-Model Alignment

Independent ASR systems can differ in transcript length and local phrasing due to insertions, deletions, and substitutions. For each clip, all model transcripts were aligned into a shared coordinate system using

Table S6. Deterministic contraction expansions used to reduce orthographic variation and support contraction-type labeling.

**Contractions → expanded forms (collapsed)**
can't→cannot; won't→will not; it's→it is; i'm→i am; you're→you are; he's→he is; she's→she is; we're→we are; they're→they are; isn't→is not; aren't→are not; don't→do not; doesn't→does not; didn't→did not; hasn't→has not; haven't→have not; hadn't→had not; wouldn't→would not; couldn't→could not; shouldn't→should not; that's→that is; what's→what is; where's→where is; who's→who is; let's→let us

Notes: Fixed *a priori*; applied after lowercasing.

Table S7. Number-word mappings used to standardize numeric expressions across ASR outputs and support numeric-type labeling.

**Mapping group**	**Source tokens**	**Normalized form**
Digits 0–10	zero, one, two, . . . , ten	0–10
Digits 11–19	eleven, twelve, . . . , nineteen	11–19
Tens	twenty, thirty, forty	20, 30, 40
Hyphenated 21–29	twenty-one, twenty-two, . . . , twenty-nine	21–29
Symbol	percent	%

Notes: Fixed *a priori*; applied case-insensitively after normalization; hyphenated forms treated as single tokens.

a progressive multiple sequence alignment (MSA) strategy at the token level. Pairwise alignments were computed using a global Needleman–Wunsch dynamic-programming algorithm with a scoring scheme designed to favor lexical matches while tolerating minor spelling variation: exact match = +10; fuzzy match = +5 (Levenshtein distance ≤ 2 for tokens longer than 2 characters); mismatch = -1; and constant gap penalty = -2. Because small edit distances can still correspond to clinically distinct medical terms, fuzzy matches were used only to improve alignment stability and not as evidence of semantic equivalence.

Multi-sequence alignment was then constructed progressively. The first transcript initialized the alignment matrix. For each subsequent transcript, a backbone sequence was extracted from the current alignment matrix by taking the first non-null token per aligned position. The new transcript was pairwise-aligned against this backbone using Needleman–Wunsch, and the alignment matrix was expanded to incorporate the new transcript by inserting null gaps where required. For reproducibility, transcript order and tie resolution were fixed deterministically. The fixed transcript insertion order for progressive alignment was: Gemini Flash 2.5, Vox Mini, Speechmatics, Whisper Large v3, Whisper Turbo v3, MedASR, NeMo QuartzNet, Wav2Vec 2.0. This choice yields a stable alignment matrix but is not guaranteed to be order-invariant; we did not perform a permutation-based sensitivity analysis in the current study. This procedure yields an aligned token matrix in which each column represents a shared aligned position, enabling per-position voting, disagreement labeling, and majority-strength computation.

5.2 S5.2 Reference Construction Modes (Consensus, Centroid, Single-Model)

Because human-verified transcripts are often unavailable in routine deployment, we evaluated cross-system agreement under three deterministic reference construction modes that define the comparator ("reference") sequence against which each ASR hypothesis is compared. All modes operate on the same aligned token matrix produced by the multi-model alignment procedure, where each row corresponds to one ASR system and each column corresponds to an aligned token position. Let $M = 8$ denote the number of ASR systems. For a given clip and aligned position j, let $t_{m,j}$ be the normalized token contributed by system m at position j, with gaps represented as $\varnothing$.

5.2.1 Consensus mode (majority-vote pseudo-reference; primary).

In Consensus mode, we construct a synthetic pseudo-reference transcript by majority voting at each aligned position j. We count the frequency of each unique non-gap normalized token across systems $\{t_{1,j}, \ldots, t_{M,j}\}$. The consensus token c_j is selected as the token with maximal frequency. Ties are resolved deterministically using a fixed system ordering: among tied tokens, the first token encountered when scanning systems in the pre-specified order is selected. If gaps constitute a strict majority at a position (i.e., $\#\{m : t_{m,j} = \varnothing\} \geq 5$ for $M = 8$), the consensus at that position is treated as a gap and the position is skipped in the final consensus string (equivalently, it is not counted among evaluated aligned positions).

This approach is conceptually aligned with classic ASR system-combination methods that align hypotheses and vote to reduce errors, such as Recognizer Output Voting Error Reduction (ROVER) (Fiscus, 1997), and with consensus/confusion-network formulations that select word sequences to minimize word-error-related objectives (Mangu et al., 2000). In our setting, lattices or confusion networks were not available from all providers, so we apply deterministic transcript-level voting on aligned token sequences. In addition, we retain per-position ensemble support by recording the *majority strength* $k_j = \#\{m : t_{m,j} = c_j\}$, yielding $k_j \in \{0, \ldots, 8\}$, which is used downstream for uncertainty visualization and risk-band mapping. Note: the per-position majority strength k_j defined here is equivalent to A_i as used in the main text; all subsequent references use A_i for consistency.

5.2.2 Denominator definitions for consensus vs HC validation.

Reference-free analyses that compute agreement, majority strength, and risk-band proportions under the Consensus pseudo-reference use the denominator "evaluated aligned positions," which excludes all-gap alignment columns and positions where the selected reference token is a gap (Table 1 in the main text). External validation against HC uses a separate denominator defined by the number of evaluated HC reference token positions after aligning ASR hypotheses to the HC transcript; this produces 76,736 evaluated positions in the present corpus. Because these denominators arise from different reference definitions and alignment scopes, they can differ slightly ($\Delta = 338$ positions), and threshold percentages (e.g., $A \leq 6$) are therefore reported under their respective denominators in the main HC section versus Table S9.

5.2.3 Centroid mode (representative real hypothesis; MBR-style).

In Centroid mode, we select a single, real system transcript that is most representative of the ensemble. For each clip, we compute pairwise token-level Levenshtein (edit) distances $d(m, n)$ between each pair of normalized transcripts. For each candidate transcript m, we compute its mean distance to all other systems:

$$\overline{d}(m) = \frac{1}{M-1} \sum_{n \neq m} d(m, n) \tag{S1}$$

The centroid reference is the transcript $m^* = \arg\min_m \overline{d}(m)$. This procedure is closely related to minimum Bayes-risk (MBR) decoding principles, where the selected hypothesis minimizes expected loss under a chosen error metric (Kumar and Byrne, 2004). In our context, centroid mode provides a robustness comparator that (i) uses an actual system output (not a synthetic reference) and (ii) is centrally located among available hypotheses, reducing dependence on an arbitrarily chosen single model.

5.2.4 Single-model reference mode.

In Single-model reference mode, one designated model output is treated as the reference sequence (after alignment and normalization), and all other systems are evaluated against it. This mode is not interpreted as ground truth. Instead, it serves as a sensitivity analysis to quantify how apparent "accuracy" and disagreement profiles can shift when a single hypothesis is privileged as the comparator in the absence of human verification, and to reveal model-dependent bias in gold-standard-free settings. In the main results, this mode was instantiated with Gemini Flash 2.5 as a strong single-model comparator because it was among the top-performing individual systems on this corpus; this choice was pragmatic and descriptive rather than a privileged reference.

5.2.5 Jackknife mode (leave-one-model-out consensus; sensitivity).

In Consensus mode, each model is compared to a pseudo-reference formed using all K hypotheses, which can introduce mild self-influence when the evaluated model contributes to the reference token at some aligned positions. To quantify the magnitude of this effect, we performed a leave-one-model-out (jackknife) variant. For each clip and evaluated model m, we construct a model-specific pseudo-reference $r^{(-m)}$ by majority voting at each aligned position using only the remaining hypotheses $\{1, \ldots, K\} \setminus \{m\}$. Ties are resolved deterministically using the same fixed system order applied in Consensus mode (excluding m). Positions where gaps constitute a strict majority among the $K - 1$ hypotheses are treated as gaps and excluded from scoring. The evaluated model m is then scored against $r^{(-m)}$ using the same token-level definitions of percent-identical and error-category rates described in S6.

5.2.6 HC annotation protocol.

HC annotation served as a pragmatic external validation layer rather than a fully independent gold-standard adjudication. For each clip, a single annotator listened to the full audio and produced a transcript based on what was heard. The consensus pseudo-reference was consulted only as a limited completeness check to ensure that no spoken segments were inadvertently omitted; it was not treated as ground truth. Corrections included insertions, deletions, and substitutions. Formatting changes (punctuation/case) were not the primary target because all analyses use normalized tokens. When audio-only disambiguation remained insufficient after repeated listening, the annotator retained the best-supported rendering using local context; in rare cases, non-identifying slide text visible in the source video was used to confirm technical terms.

6 SUPPLEMENTARY METHOD S6. ERROR TAXONOMY AND DERIVATION FROM ALIGNED TOKENS

For each clip and evaluation instance (i.e., each reference construction mode), we compared every model hypothesis to the selected reference token-by-token using the aligned transcript matrix. Let r_i and h_i denote the reference and hypothesis entries at aligned position i, where either entry may be a gap. All comparisons were performed on the normalized token representation.

Each evaluated position was assigned exactly one error label using a deterministic decision order, yielding a mutually exclusive taxonomy. Positions where both entries were gaps ($r_i = \varnothing$ and $h_i = \varnothing$) were excluded from scoring. For all remaining positions, labels were assigned in the following order: (1) *Identical* ($h_i = r_i$ after normalization), (2) *Punctuation*, (3) *Contraction*, (4) *Numeric*, (5) *Filler*, (6) *Content* (all remaining lexical mismatches, including meaning-bearing differences; this category subsumes substitution-, insertion-, and deletion-type discrepancies under the alignment). At the clip $\times$ model level, we recorded per-category

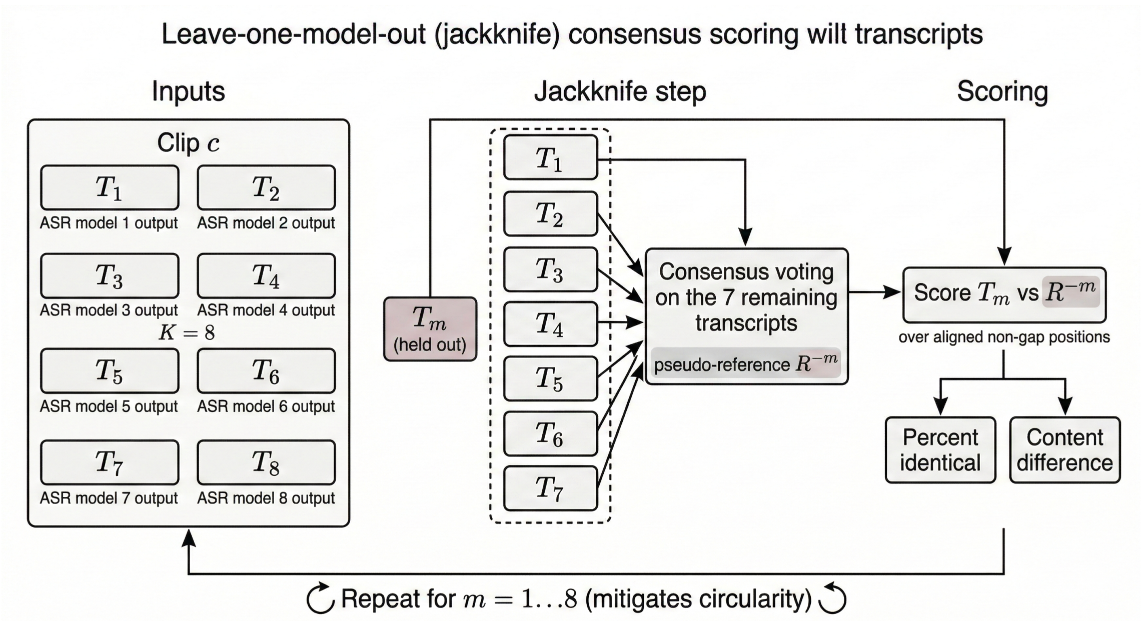

Figure S1. Leave-one-model-out (jackknife) consensus scoring for per-model evaluation ($K = 8$). For each clip c, eight ASR transcripts $\{T_1, \dots, T_8\}$ are available. To score a given model m, its transcript T_m is held out and a pseudo-reference $R^{(-m)}$ is constructed by applying the same consensus voting rule to the remaining $K - 1 = 7$ transcripts $\{T_j : j \neq m\}$. Model T_m is then compared to $R^{(-m)}$ over aligned non-gap positions to compute agreement metrics (percent identical and content difference). This procedure is repeated for $m = 1, \dots, 8$ to mitigate circularity when evaluating a model against an ensemble-derived pseudo-reference.

counts $N_{\text{ident}}, N_{\text{punct}}, N_{\text{contr}}, N_{\text{num}}, N_{\text{fill}}, N_{\text{content}}$, along with total tokens N_{total} and total difference N_{diff}. Here, N_{total} denotes the number of evaluated aligned positions (all positions except gap–gap), and

$$N_{\text{diff}} = N_{\text{total}} - N_{\text{ident}} = N_{\text{punct}} + N_{\text{contr}} + N_{\text{num}} + N_{\text{fill}} + N_{\text{content}}. \tag{S2}$$

We enforced arithmetic consistency checks (e.g., the identity above) to ensure all downstream rates are well-defined and auditable.

7 SUPPLEMENTARY METHOD S7. STATISTICAL FORMULAS AND DERIVATIONS

7.1 S7.1 Intraclass Correlation Coefficient (ICC[2,1], Absolute Agreement)

Inter-model reliability was quantified using the two-way random-effects, single-measure intraclass correlation coefficient for absolute agreement, ICC(2,1), treating clips as targets and ASR systems as raters. Let K denote the number of systems (raters) and C the number of clips (targets). ICC(2,1) can be expressed using mean squares from a two-way random-effects ANOVA as:

$$\text{ICC}(2,1) = \frac{MS_{\text{targets}} - MS_{\text{error}}}{MS_{\text{targets}} + (K-1)MS_{\text{error}} + \frac{K}{C}(MS_{\text{raters}} - MS_{\text{error}})} \tag{S3}$$

where MS_{targets} is the mean square for targets (clips), MS_{raters} is the mean square for raters (ASR systems), and MS_{error} is the residual mean square, $n = 50$ clips, and $K = 8$ models. ICC was computed separately

for $p_{\text{identical}}$ and r_{content} using the clip-level score matrices described in the main Methods (Shrout and Fleiss, 1979; McGraw and Wong, 1996; Koo and Li, 2016).

7.2 S7.2 Majority-Strength Counts and Risk-Band Proportions

Let K denote the number of ASR systems included in the evaluation and let M_c denote the number of evaluated aligned positions for clip c (excluding all-gap columns and excluding positions where the reference token is a gap, consistent with the UI display). For each evaluated position $i \in \{1, \ldots, M_c\}$, define $A_{c,i} \in \{0, \ldots, K\}$ as the number of systems whose normalized token matches the reference token at that position (systems contributing a gap are treated as non-matches).

The agreement histogram for clip c is:

$$n_k(c) = |\{i : A_{c,i} = k\}|, \qquad \sum_{k=0}^{K} n_k(c) = M_c \tag{S4}$$

with proportions $n_k(c)/M_c$. From this histogram, we report:

$$p_{\text{full}}(c) = \frac{n_K(c)}{M_c}, \quad p_{\text{strong}}(c) = \frac{n_{K-1}(c) + n_K(c)}{M_c}, \quad p_{\text{split}}(c) = 1 - p_{\text{strong}}(c) = \frac{\sum_{k=0}^{K-2} n_k(c)}{M_c} \tag{S5}$$

7.2.0.1 Visualization risk bands (three-bin summary).

For visualization, majority strength can also be grouped into low/medium/high-risk bands: Low-risk: $A_{c,i} \in \{7, 8\}$; Medium-risk: $A_{c,i} \in \{4, \ldots, 6\}$; High-risk: $A_{c,i} \in \{0, 1, 2, 3\}$.

Clip-level band proportions are computed as the fraction of evaluated positions falling in each set (with denominator M_c). Corpus-level summaries are computed by aggregating indicators across clips using $M_{\text{total}} = \sum_c M_c$ as the denominator.

7.3 S7.3 Spearman Rank Correlation

For each model pair (A, B), we formed clip-wise score vectors (e.g., $p_{\text{identical}}(c, A)$ and $p_{\text{identical}}(c, B)$, $c = 1, \ldots, C$) and computed Spearman's rank correlation coefficient ρ to quantify monotonic association in clip-level behavior. Spearman correlations and two-sided p-values were obtained using `scipy.stats.spearmanr`. Prior to analysis, paired observations were filtered to remove non-finite values (NaN/Inf). Ties were handled using standard rank assignment as implemented in SciPy.

To quantify uncertainty in ρ, we estimated 95% confidence intervals using a nonparametric bootstrap percentile procedure over clips. Specifically, we resampled paired clip observations $\{(x_c, y_c)\}_{c=1}^{n}$ with replacement to generate $B = 10{,}000$ bootstrap samples of size n (where n is the number of valid clips after filtering). For each bootstrap sample, we recomputed ρ, yielding an empirical bootstrap distribution of ρ. The 95% confidence interval was defined by the 2.5th and 97.5th percentiles of this distribution. A fixed random seed (42) was used to ensure reproducibility.

7.4 S7.4 Mean Paired Differences (Systematic Offsets)

To quantify systematic performance offsets not captured by correlation, we computed the mean paired difference in clip-level percent-identical (percentage points) for each model pair:

$$\Delta_{A,B}^{(p)} = \frac{1}{C} \sum_{c=1}^{C} \left(p_{\text{identical}}(c, A) - p_{\text{identical}}(c, B) \right) \tag{S6}$$

An analogous definition can be applied to r_{content} when systematic differences in content-difference rates are of interest.

7.5 S7.5 Content Enrichment Across Disagreement Strata

For analyses that stratify clips by disagreement mass (e.g., p_{split} or a high-risk band proportion), category fractions were computed as:

$$f_{\text{content}}(c) = \frac{N_{\text{content}}(c)}{N_{\text{diff}}(c)}, \quad f_{\text{punct}}(c) = \frac{N_{\text{punct}}(c)}{N_{\text{diff}}(c)}, \quad \ldots \tag{S7}$$

where $N_{\text{diff}}(c)$ is the total number of labeled differences in clip c (across all categories). Associations between disagreement mass and category fractions were assessed using Spearman correlation, consistent with the main Statistical Analysis.

8 SUPPLEMENTARY FIGURE S2

Each clip was assigned a "winner" ASR model defined as the system achieving the highest percent-identical score ($p_{\text{identical}}$) relative to the Consensus pseudo-reference. The figure summarizes winner counts stratified by accent group. Bars represent accent groups; stacked segments indicate the number of clips won by each model within that accent group. The plot provides a descriptive summary of winner counts by accent group; because many groups contain a single clip, it should not be interpreted as evidence of stable accent-specific winner patterns.

9 SUPPLEMENTARY TABLE S8

10 SUPPLEMENTARY TABLE S9

REFERENCES

Fiscus, J. G. (1997). A post-processing system to yield reduced word error rates: Recognizer Output Voting Error Reduction (ROVER). In *1997 IEEE Workshop on Automatic Speech Recognition and Understanding Proceedings* (Santa Barbara, CA, USA: IEEE), 347–354. doi:10.1109/ASRU.1997.659110

Koo, T. K. and Li, M. Y. (2016). A guideline of selecting and reporting intraclass correlation coefficients for reliability research. *Journal of Chiropractic Medicine* 15, 155–163

Kumar, S. and Byrne, W. (2004). Minimum Bayes-risk decoding for statistical machine translation. In *Proceedings of the Human Language Technology Conference of the North American Chapter of the Association for Computational Linguistics (HLT-NAACL)*. 458–465

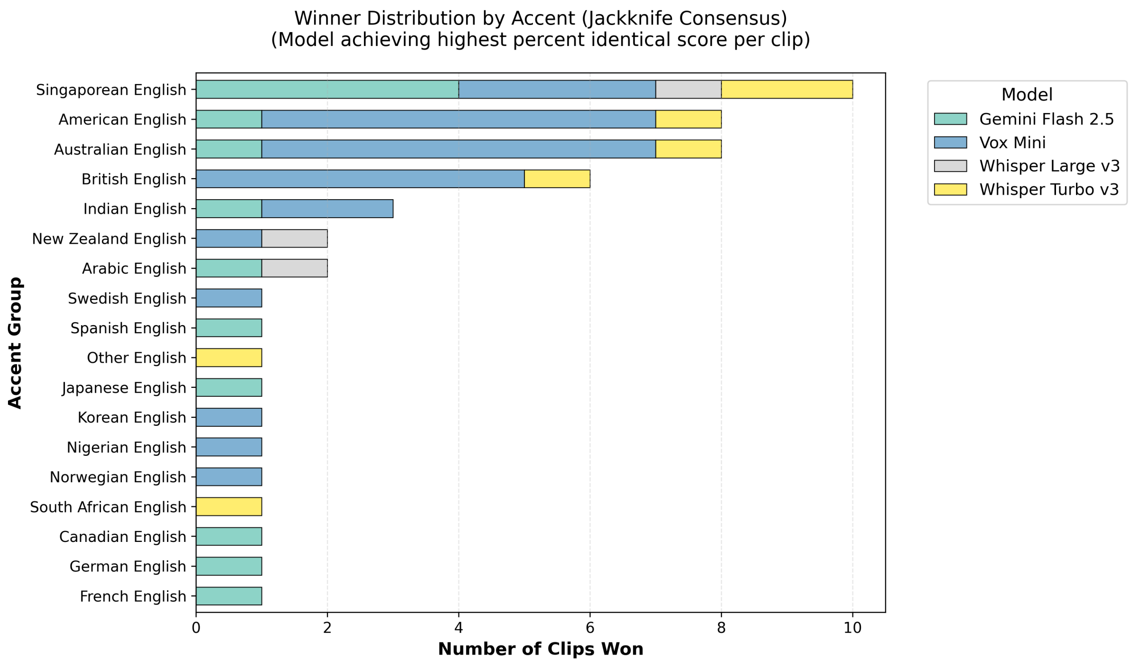

Figure S2. Winner model by accent group under jackknife scoring (leave-one-model-out consensus pseudo-reference).

Mangu, L., Brill, E., Stolcke, A., et al. (2000). Finding consensus in speech recognition: word error minimization and other applications of confusion networks. *Computer Speech & Language* 14, 373–400. doi:10.1006/csla.2000.0152

McGraw, K. O. and Wong, S. P. (1996). Forming inferences about some intraclass correlation coefficients. *Psychological Methods* 1, 30–46

Shrout, P. E. and Fleiss, J. L. (1979). Intraclass correlations: uses in assessing rater reliability. *Psychological Bulletin* 86, 420–428

Table S8. Majority-strength confidence bands by accent group ($K = 8$; Consensus mode).

Accent (English)	n_{clips}	n_{tokens}	High-risk % (0–3)	Medium-risk % (4–6)	Low-risk % (7–8)
Singaporean	10	15,614	2.57	28.28	69.15
American	8	12,973	1.82	21.58	76.60
Australian	8	12,207	1.38	22.71	75.91
British	6	9,911	1.46	22.80	75.73
Indian	3	4,633	4.14	24.43	71.42
Arabic	2	2,352	3.27	24.74	71.98
New Zealand	2	3,646	1.78	20.38	77.84
French	1	952	6.20	31.62	62.18
Filipino	1	573	0.70	28.27	71.03
Canadian	1	1,936	3.10	21.95	74.95
Japanese	1	1,028	5.74	40.56	53.70
German	1	1,060	5.38	31.98	62.64
Nigerian	1	2,158	4.12	45.74	50.14
Korean	1	1,144	4.72	29.90	65.38
Norwegian	1	1,472	1.56	20.79	77.65
South African	1	2,117	3.54	35.76	60.70
Spanish	1	901	11.43	44.73	43.84
Swedish	1	1,721	0.81	16.15	83.03

Note: n_{clips} denotes the number of clips in the accent group, and n_{tokens} denotes the number of evaluated aligned positions contributing to the distribution. For each evaluated position i, majority strength $A_i \in \{0, \ldots, 8\}$ is the number of ASR systems whose normalized token matches the consensus pseudo-reference token at that position (models contributing a gap are treated as non-matches). Risk bands are defined as: high-risk ($A_i \in \{0, 1, 2, 3\}$), medium-risk ($A_i \in \{4, 5, 6\}$), and low-risk ($A_i \in \{7, 8\}$). Percentages are computed within accent group and sum to 100%. Accent-stratified results are exploratory given small group sizes for several accents.

Table S9. Corpus-level threshold sweep for majority-strength review thresholds ($K = 8$; Consensus mode).

Threshold	Reviewed (%)	Work reduction (%)	Avg disagreeing models (reviewed)	Enrichment (× baseline)	Disagr. mass captured (%)
$A \leq 3$	2.46	97.54	5.21	5.18×	12.76
$A \leq 4$	7.94	92.06	4.37	4.35×	34.56
$A \leq 5$	17.47	82.53	3.62	3.61×	62.99
$A \leq 6$	27.89	72.11	3.02	3.00×	83.71

Note: Reviewed (%) is the fraction of evaluated aligned positions with majority strength $A_i \leq t$. Work reduction (%) = 100− Reviewed (%). Avg disagreeing models within reviewed is the conditional mean of $(K - A_i)$ among reviewed positions. Disagreement enrichment is the ratio of this conditional mean to the corpus-wide mean of $(K - A_i)$. Disagreement mass captured (%) is the fraction of total disagreement mass (sum of $K - A_i$ over all positions) contained within the reviewed subset. Enrichment is type-agnostic (all mismatch types); content-only enrichment would require token-level error typing within each A-threshold. The highlighted thresholds discussed in the main text ($A \leq 5$ and $A \leq 6$) are illustrative post hoc operating points, not pre-registered decision thresholds. Percentages in this table use the Consensus-mode denominator (evaluated aligned positions; Table 1 in the main text); therefore the reviewed fraction at a given threshold (e.g., $A \leq 6 = 27.89\%$) can differ slightly from the HC workload–recall analysis, which uses the HC alignment denominator (e.g., $A \leq 6 = 28.6\%$).
